# Synthesis of Novel Planar-Chiral Charge-Compensated *nido*-Carborane-Based Amino Acid

**DOI:** 10.3390/molecules29184487

**Published:** 2024-09-21

**Authors:** Dmitry A. Gruzdev, Angelina A. Telegina, Marina A. Ezhikova, Mikhail I. Kodess, Galina L. Levit, Victor P. Krasnov

**Affiliations:** Postovsky Institute of Organic Synthesis, Russian Academy of Sciences (Ural Branch), Ekaterinburg 620108, Russia; telegina@ios.uran.ru (A.A.T.); ema@ios.uran.ru (M.A.E.); nmr@ios.uran.ru (M.I.K.); ca512@ios.uran.ru (G.L.L.); ca@ios.uran.ru (V.P.K.)

**Keywords:** alkylation, amino acid, diastereomers, 7,8-dicarba-*nido*-undecaborane, NMR spectral assignment, planar chirality, zwitterionic compounds

## Abstract

Amino acids with unusual types of chirality and their derivatives have recently attracted attention as precursors in the synthesis of chiral catalysts and peptide analogues with unique properties. In this study, we have synthesized a new *nido*-carborane-based planar-chiral amino acid, in the molecule of which the amino group is directly bonded to the B(3) atom, and the carboxyl group is attached to the B(9) atom through the CH_2_S^+^(Me) fragment. 3-Amino-9-dimethylsulfonio-*nido*-carborane, prepared in three steps from 3-amino-*closo*-carborane in a high yield, was a key intermediate in the synthesis of the target planar-chiral amino acid. The carboxymethyl group at the sulfur atom was introduced by the demethylation reaction of the dimethylsulfonio derivative, followed by S-alkylation. The structure of new 3,9-disubstituted *nido*-carboranes was studied for the first time using NMR spectroscopy. The resonances of all boron atoms in the ^11^B NMR spectrum of 3-amino-9-dimethylsulfonio-*nido*-carborane were assigned based on the 2D NMR correlation experiments. The *nido*-carborane-based planar-chiral amino acid and related compounds are of interest as a basis for peptide-like compounds and chiral ligands.

## 1. Introduction

Chirality is a unique property of matter that underlies molecular recognition and life itself [1,2,3,4]. To date, significant progress has been achieved in the preparation of organic and organoelement compounds with different types of chirality (central, axial, planar, etc.) [5,6,7,8,9,10].

Amino acids are one of the most important groups of chiral compounds. As a rule, natural amino acids contain an asymmetric carbon atom at position 2. Derivatives of amino acids with the property of central chirality are widely used in the design of medicinal agents, functional materials, and chiral catalysts [11,12,13,14,15,16].

**Figure 1:**
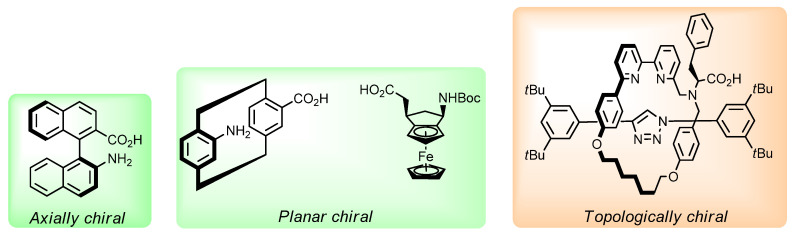
Selected examples of amino acid derivatives possessing unusual forms of chirality.

Recently, the preparation and application of compounds (including amino acid derivatives) possessing unusual types of chirality have attracted more and more attention (Figure 1). Synthetic approaches to racemic and enantiomerically pure derivatives and analogs of amino acids possessing axial (biphenyl and binaphthyl derivatives [17,18,19,20,21,22,23,24,25,26]), planar (ferrocene derivatives [27,28,29,30], [2.2]paracyclophane [31,32,33,34,35,36] and macrocyclic cyclophanes [37,38,39]), and helical chirality [40,41,42,43,44] have been proposed. Amino acid derivatives containing adamantane [45,46,47], calix[4]arene [48,49], and fullerene [50,51,52] residues as a chirality element, as well as catenane and rotaxane motifs possessing topological chirality [53,54,55], have been synthesized. It has been shown that the introduction of amino acid fragments allows obtaining stable conformational isomers of pillar[5]arenes [56,57,58,59]. Axial and planar-chiral derivatives of amino acids find application in chiral organocatalysis [19,20,21,23,34,60,61,62,63] and the design of peptides with original structures [28,64].

**Figure 2:**
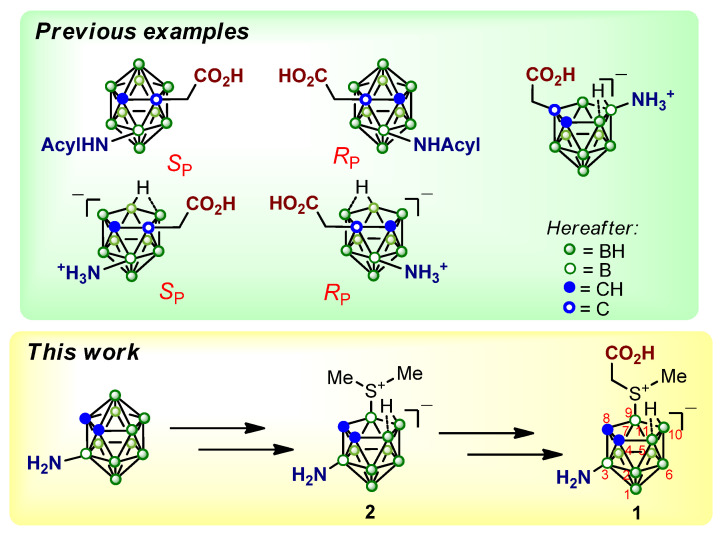
Planar-chiral carborane-based amino acids.

A special group of unnatural amino acids is represented by derivatives of 1,2-dicarba-*closo*-dodecaborane and 7,8-dicarba-*nido*-undecaborane (carboranes). Molecules of *ortho*-*closo*- and *nido*-carboranes have an icosahedral structure and contain ten or nine boron atoms, respectively, and two carbon atoms located at adjacent vertices of the cluster [65,66]. Carborane derivatives are of interest primarily as potential agents for boron delivery in boron neutron capture therapy (BNCT) of tumors [67,68,69,70,71], as well as in combined approaches such as BNCT/GdNCT [72,73,74]. Carborane-based amino acids, in the molecules of which the carboxyl and amino groups are attached to different cluster vertices, are planar chiral [75]. We have developed methods for the preparation of a number of planar-chiral amino acid derivatives based on *closo*- [76,77,78] and *nido*-carboranes [79,80] (Figure 2), methods for analyzing their stereoisomeric composition and carried out the assignment of the absolute configuration. It has been shown that crystals of planar-chiral *closo*-carborane–based *pseudo*-peptides can have significant piezoelectric activity [77,81].

Recently, we have synthesized a series of carborane-containing derivatives of natural amino acids in which the *nido*-carborane residue carries a negative charge, and the positive charge is localized on the nitrogen or sulfur atoms [82,83,84]. In these sulfur-containing α-amino acids, a *nido*-carborane residue is located in the side chain [83,84]. A number of primary amines and carboxylic acids were obtained earlier on the basis of 9-methylthio-*nido*-carborane [85]. In the present work, we synthesized for the first time a planar-chiral *nido*-carborane-based amino acid **1** (Figure 2), in which the amino group is attached to the B(3) atom, and the carboxyl group is linked to the B(9) position via the sulfur atom carrying a positive charge. Using the example of dimethylsulfonio derivative **2**, the precursor of amino acid **1**, we have, for the first time, carried out a detailed NMR spectroscopy study of the structure of a *nido*-carborane derivative containing amino and dialkysulfonio substituents at positions 3 and 9.

## 2. Results and Discussion

**Scheme 1:**
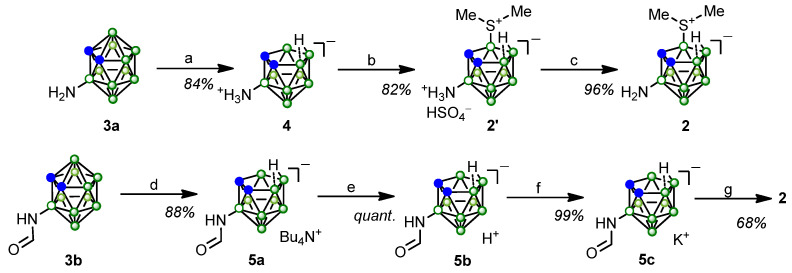
Synthesis of 3-amino-9-dimethylsulfonio-*nido*-carborane (**2**). (a) piperidine, benzene, rt, 48 h [86]; (b) DMSO, H_2_SO_4_, H_2_O, 80 °C, 4.5 h; (c) NaHCO_3_, H_2_O, rt, 0.5 h; (d) TBAF, THF/H_2_O, rt, 9 days; (e) Amberlite IR-120 (H form), EtOH, rt, 1 h; (f) 1. KOH, EtOH, 0.5 h; 2. CO_2_ solid (excess), rt, 0.5 h; (g) 1. DMSO, H_2_SO_4_, H_2_O, 80 °C, 4.5 h; 2. KOH, rt, 1 h.

The 3-amino-*nido*-carborane derivative **2** is a key intermediate in the synthesis of planar-chiral amino acid **1**. We studied the possibilities of obtaining compound **2** starting from 3-amino-*closo*-carborane (**3a**) and its *N*-formyl derivative **3b** (Scheme 1).

Deboronation of compound **3a** according to the known method [86] smoothly led to 3-ammonio-*nido*-carborane (**4**). Treatment of compound **4** with dimethyl sulfoxide (DMSO) in the presence of sulfuric acid resulted in the corresponding 9-dimethylsulfonio derivative in the form of a salt with sulfuric acid (**2′**) (Scheme 1). Previously, the introduction of the dimethylsulfonio group by an acid-promoted reaction with DMSO was carried out only at position 9 of unsubstituted *nido*-carborane [87,88,89] or *nido*-carborane with substituents at carbon atoms [90,91,92]. In our case, compound **4** with an amino group at position 3 of *nido*-carborane was highly reactive, and compound **2′** was isolated in high yield.

The structure of compound **2′** was confirmed by NMR spectroscopy and chromato-mass spectrometry. In the ^1^H NMR spectrum recorded in DMSO-*d*_6_ (Figure S1), we observed a set of signals characteristic of 9-substituted *nido*-carboranes: a broadened signal in the region δ −3.1…−3.0 ppm corresponding to the bridging hydrogen atom and singlets at δ 2.37 and 3.04 ppm, corresponding to nonequivalent CH protons of carborane. The protons of the S(Me)_2_ diastereotopic methyl groups resonated at δ 2.65 and 2.77 ppm, respectively. In the ^11^B NMR spectrum of compound **2′** (Figure S2), there were nine separate peaks, which indicated an asymmetric structure of the molecule and pointed to the presence of a substituent at position 9. The resonances of two nonequivalent carbon atoms of 9-substituted *nido*-carborane in the ^13^C NMR spectrum (Figure S3) were in the characteristic region (broadened peaks at δ 41 and 54 ppm, respectively).

Neutralization of salt **2′** with aqueous NaHCO_3_ solution afforded 3-amino derivative **2** in high yield.

An alternative method for the synthesis of compound **2** starting from 3-formamido-*closo*-carborane (**3b**) involved its deboronation followed by the introduction of a dimethylsulfonio group. The interaction of the *closo*-derivative **3b** with tetrabutylammonium fluoride (TBAF) at room temperature, by analogy with the approach proposed by us earlier [93], proceeded regioselectively and led to the *nido*-derivative **5a** in high yield (Scheme 1). Treatment of tetrabutylammonium salt **5a** with a strongly acidic ion exchange resin afforded the protonated *nido*-carborane **5b**, which was further converted to the corresponding potassium salt **5c**. Potassium salt **5c** was highly hygroscopic and prone to solvate with ethanol; even after drying in vacuo at 70 °C for 6 h, the ethanol content was 50 mol% (according to ^1^H NMR spectroscopy). The reaction of the formyl derivative **5c** with DMSO in aqueous sulfuric acid resulted in the simultaneous introduction of a dimethylsulfonio group into position 9 of *nido*-carborane and hydrolysis of the formamido group, thus yielding compound **2′** as the only reaction product. After adjusting the reaction mixture with potassium hydroxide to pH ~ 8, subsequent extraction with EtOAc, and chromatographic purification, free 3-amino-9-dimethylsulfonio-*nido*-carborane (**2**) was isolated in 68% yield.

In the NMR spectra of compounds **5a**–**c** (DMSO-*d*_6_), we observed double sets of signals corresponding to *trans*- and *cis*-isomers due to hindered rotation around the amide bond (Figures S4–S12). A rotamer ratio of approximately 85:15 is typical for secondary formamides [94,95,96].

**Figure 3:**
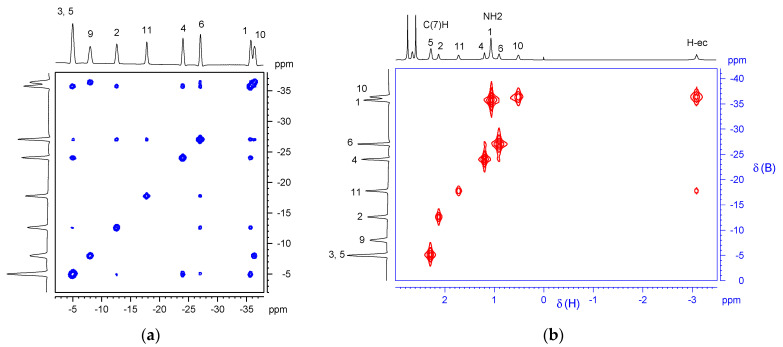
2D NMR spectra of compound **2**: (**a**) ^11^B–^11^B COSY spectrum (160 MHz, CDCl_3_); (**b**) 2D ^1^H{^11^B}–^11^B{^1^H} HMQC spectrum (500 MHz, CDCl_3_).

The structure of 3-amino-9-dimethylsulfonio-*nido*-carborane (**2**) was studied using a combination of 2D NMR experiments: ^11^B–^11^B{^1^H} COSY, ^1^H–^1^H{^11^B} COSY, ^1^H–^13^C HSQC and ^1^H{^11^B}–^11^B HMQC (Figure 3). ^1^H NMR spectrum of compound **2** was recorded with broad-band ^11^B-decoupling. Analysis of the correlation spectra allowed, for the first time, a complete and unambiguous assignment of the ^1^H, ^11^B, and ^13^C resonances of the 3,9-disubstituted *nido*-carborane derivative. It is significant that in the ^1^H{^11^B}–^11^B HMQC spectrum, the bridging “extra” (endocyclic) proton H-*ec*, located above the open pentagonal plane C_2_B_3_, has the most intense cross-peak with the B-10 boron, which indicates its probable localization near this atom.

**Scheme 2:**
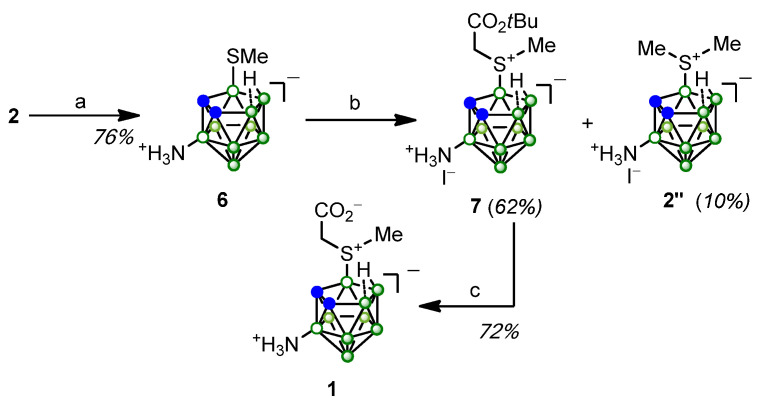
Synthesis of planar chiral *nido*-carborane-based amino acid **1**: (a) naphthalene, Na, THF, 10 °C → rt, 7 days; (b) ICH_2_COO*t*Bu, EtOH, Δ, 10 h; (c) CF_3_COOH, CH_2_Cl_2_, rt, 3 h.

Demethylation of compound **2** with sodium naphthalene by analogy with the known approach [83,97] smoothly led to 3-ammonio-9-methylthio-*nido*-carborane (**6**) as an internal salt in good yield (Scheme 2). Compound **6** contains a chiral plane in the structure, so it is a mixture of two enantiomers (1:1) distinguishable by chiral HPLC (Figure S33). Zwitterion **6** is a valuable building block from which various charge-compensated planar-chiral derivatives of 3-amino-*nido*-carborane can be prepared.

Alkylation of thioether **6** with *tert*-butyl iodoacetate led to the 3-ammonio-*nido*-carboran-9-yl derivative of *S*-methylthioglycolic acid as an iodide (compound **7**), in the molecule of which the *nido*-carborane cluster is linked to positively charged nitrogen and sulfur atoms. A by-product, 3-amino-9-dimethylsulfonio-*nido*-carborane as a hydroiodide (**2″**), was isolated in 10% yield. Cleavage of *tert*-butyl ester group in compound **7** under the action of trifluoroacetic acid followed by crystallization and chromatographic purification successively led to compound **1** containing positively charged nitrogen and sulfur atoms along with carboxylate group and *nido*-carborane cage bearing negative charges.

From the literature, it is known that sulfonium derivatives can be highly reactive alkylating and arylating agents [98,99,100]. We have previously noted the instability of dialkylsulfonio *nido*-carborane derivatives under basic conditions [83]. Examples of the formation of dimethylsulfonio-*nido*-carborane as a by-product in the alkylation reactions of the corresponding methylthio derivative were reported [101], but the mechanism of this process has not been studied. It has been shown that dialkylsulfonio dodecaborane derivatives exhibit alkylating ability, including when interacting with methylthio-dodecaborane [102,103]. In our case, the formation of compound **2″** apparently occurs as a result of the transfer of a methyl group from the molecule of the main reaction product **7** to the molecule of the starting thioether **6**.

**Scheme 3:**
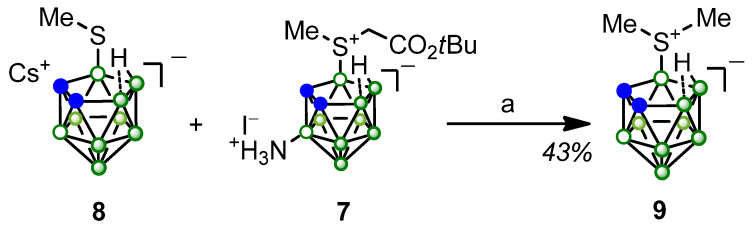
Interaction of compound **7** with 9-methylthio-*nido*-carborane cesium salt (**8**): (a) EtOH, Δ, 28 h.

The alkylating ability of compound **7** was illustrated in a model reaction with the cesium salt of 9-methylthio-7,8-dicarba-*nido*-undecaborane (**8**) (Scheme 3). Keeping an equimolar mixture of compounds **7** and **8** in refluxing ethanol for 28 h resulted in the formation of a complex mixture of transalkylation products. We managed to isolate the predominant product, 9-dimethylsulfonio-7,8-dicarba-*nido*-undecaborane (**9**), in pure form from the reaction mixture. The formation of compound **9** provides clear evidence that compound **7** is prone to demethylation in the presence of suitable nucleophiles.

The negative charge of the *nido*-carborane cluster in compounds **1**, **2′**, **2″**, and **7** is compensated by the positive charge localized on the sulfur atom. The amino group at position 3 of *nido*-carborane is prone to protonation; the counterion, in this case is hydrosulfate or iodide anions. Examples of stable *nido*-carborane derivatives containing two substituents bearing a positive charge at different vertices of the cluster are very rare [91,104]. The derivatives we obtained were stable when stored in solid form for more than 2 months but were prone to decomposition during chromatographic purification on silica gel and storage in DMSO solutions.

The structure of the obtained compounds was proved by ^1^H, ^11^B, and ^13^C NMR spectroscopy and high-resolution mass spectrometry. Compounds **1** and **7**, containing a chiral plane and an asymmetric sulfur atom in their structure, are mixtures of four diastereomers with similar physicochemical properties. We failed to select the conditions for their HPLC separation in various normal and reversed-phase modes.

**Scheme 4:**
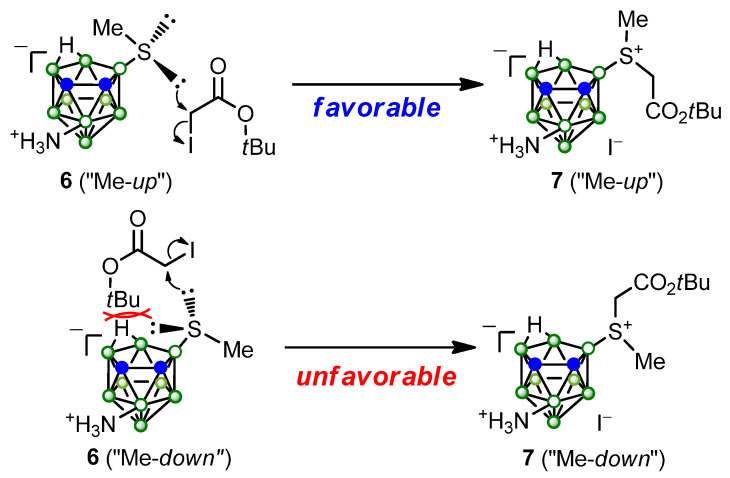
Possible orientations of SMe group in compound **6** leading to the formation of different diastereomers of compound **7** (only compounds of (*R*_P_)-configuration are shown).

The ratio of the integral intensities of the proton signals of the diastereomers of compounds **1** and **7** in the ^1^H NMR spectra was 7:3 and 6:4, respectively, which is consistent with the previously observed formation of diastereomers of 9-dialkylsulfonio *nido*-carboranes in nonequivalent amounts [84,101,105]. Apparently, this phenomenon is due to the fact that the lone pair of electrons of the sulfur atom in thioether **6** is more accessible for interaction with the alkylating agent in the case when the methyl group is turned “upwards” with respect to the “open pentagonal face” of *nido*-carborane, and lone pair of electrons of sulfur atom is not shielded by a high electronic density near the open face of *nido*-carborane (Scheme 4). This results in the preferential formation of isomer **7**, in which the *tert*-butoxycarbonylmethyl substituent occupies a “down-directed position” relative to the “open face” of the carborane, and the methyl substituent is in an “up-directed position”. The formation of diastereomers of compound **7** in nonequivalent amounts may also be due to the fact that one of them is more thermodynamically stable than the other. 

**Figure 4:**
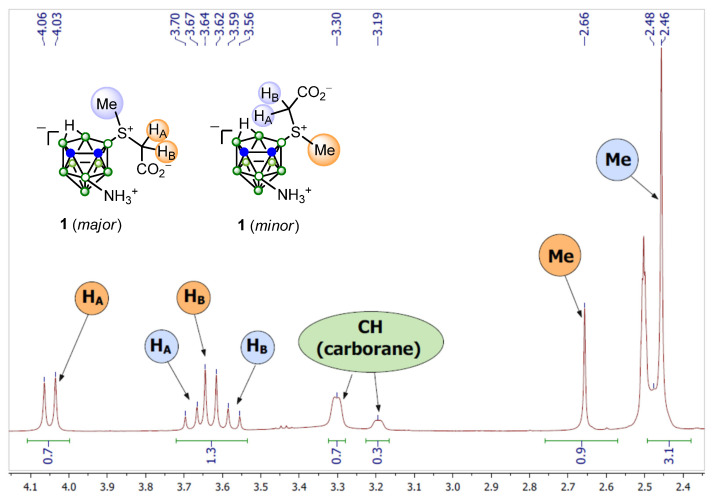
Fragment of ^1^H NMR spectrum of compound **1**.

Accordingly, in the ^1^H NMR spectra, the methylene protons of the predominant diastereomers of compounds **1** and **7** are down-field shifted as compared to the resonances corresponding to the minor diastereomers; the peaks of the methyl group protons of the predominant diastereomers are up-field shifted (Figure 4). This is due to the presence of an induced diatropic ring current in the *nido*-carborane cage, which causes an up-field shift of the endocyclic proton and apical boron-1 [106].

## 3. Materials and Methods

3-Ammonio-7,8-dicarba-*nido*-undecaborane (**4**) [86], 3-formamido-1,2-dicarba-*closo*-dodecaborane (**3b**) [107], cesium salt of 9-methylthio-7,8-dicarba-*nido*-undecaborane (**8**) [83] and *tert*-butyl iodoacetate [108] were obtained according to known procedures. Other reagents were commercially available and were purchased from Alfa Aesar (Heysham, UK). Solvents were purified according to traditional methods [109] and used freshly distilled. Melting points were obtained on a SMP3 apparatus (Barloworld Scientific, Staffordshire, UK). The ^1^H, ^11^B, and ^13^C NMR spectra were recorded on Bruker DRX-400 (operating frequencies of 400 (^1^H) and 128 (^11^B) MHz), Bruker AVANCE 500 (operating frequencies of 500 (^1^H), 160 (^11^B), and 126 (^13^C) MHz) or Bruker AVANCE NEO 600 (operating frequencies of 193 (^11^B) and 151 (^13^C) MHz) instruments (Bruker, Karlsruhe, Germany) at ambient temperature using TMS as an internal standard and BF_3_·Et_2_O as an external standard. ^1^H NMR spectrum of compound **2** was recorded with broad-band ^11^B-decoupling. For compound **2**, signals in ^1^H, ^11^B and ^13^C spectra were assigned based on 2D ^1^H–^1^H{^11^B} and ^11^B–^11^B{^1^H} COSY, ^1^H–^13^C HSQC and ^1^H{^11^B}–^11^B HMQC experiments. NMR spectra of the compounds obtained, see the Supplementary Materials, Figures S1–S32. Analytical TLC was performed using Sorbfil plates (Imid, Krasnodar, Russia); visualization under UV light (254 nm) and by treatment with a 0.75% KMnO_4_ solution in 0.016 M NaOH solution containing 5% K_2_CO_3_. Flash column chromatography was performed using Silica gel 60 (230–400 mesh) (Alfa Aesar, Heysham, UK). The high-resolution mass spectra were obtained on a Bruker maXis Impact HD mass spectrometer (Bruker, Karlsruhe, Germany), electrospray ionization or chemical ionization at atmospheric pressure in positive or negative mode with direct sample inlet (4 L/min flow rate). Analytical HPLC of compound **6** was carried out on an AZURA instrument (KNAUER, Berlin, Germany) using a Chiralpak AS-H column (250 × 4.6 mm, 5 µm) (Daicel Corp., Osaka, Japan); flow rate 1.0 mL/min, detection at 220 nm, *n*-hexane–*i*PrOH 5:1 as an eluent. HPLC of compound **6**, see the Supplementary Materials, Figure S33.

*3-Ammonio-9-dimethylsulfonio-7,8-dicarba-nido-undecaborane Hydrosulfate* (**2′**). Water (0.48 mL) and H_2_SO_4_ conc. (0.60 mL) were slowly added to a solution of compound **4** (0.063 g, 0.42 mmol) in DMSO (0.13 mL) under argon atmosphere under stirring at room temperature. The reaction mixture was stirred at 80 °C for 4.5 h, then cooled to room temperature. Water (5 mL) and solid KOH were added to the reaction mixture to pH ~ 7, and then EtOH (7 mL) was added. The precipitate was filtered off and washed with EtOH (3 mL). The filtrate and washings were evaporated to dryness under reduced pressure. The residue was purified by flash chromatography on silica gel (eluent CHCl_3_–EtOH from 8:2 to 4:6). Yield 0.11 g (82%). Light beige powder, m.p. 217–222 °C (decomp.). TLC (CHCl_3_–EtOH–AcOH 5:5:0.1): *R*_f_ 0.17. ^1^H NMR (DMSO-*d*_6_, 400 MHz) δ (ppm): −3.08 (br. s, 1H, BHB), 0.0–2.4 (br. m, 7H, 7 × BH), 2.37 (s, 1H, CH-carborane), 2.65 (s, 3H, Me), 2.77 (s, 3H, Me), 3.04 (s, 1H, CH-carborane), 7.05 (br. s, 3H, NH_3_^+^). ^11^B NMR (DMSO-*d*_6_, 193 MHz) δ (ppm): −37.4 (d, *J* = 138.5 Hz, 1B), −34.4 (br. s, 1B), −26.8 (d, *J* = 145.5 Hz, 1B), −23.9 (d, *J* = 132.6 Hz, 1B), −18.2 (1B), −16.7 (br. s, 1B), −13.8 (br. s, 1B), −10.7 (s, 1B), −5.5 (br. m, 2B). ^13^C NMR (DMSO-*d*_6_, 151 MHz) δ (ppm): 25.3 (Me), 27.1 (Me), 41.4 (CH-carborane), 54.4 (CH-carborane). HRMS (ESI) (*m*/*z*) [M − HSO_4_]^+^: calcd. for [C_4_H_19_^11^B_9_NS]^+^: 212.2079, found: 212.2081.

*3-Formamido-7,8-dicarba-nido-undecaborane Tetrabutylammonium Salt* (**5a**). A mixture of compound **3b** (0.59 g, 3.15 mmol), 75% aqueous TBAF solution (5.75 mL, 15.75 mmol), and THF (16 mL) was kept at room temperature for 9 days, then EtOAc (50 mL) was added, and the mixture was washed successively with water (3 × 25 mL) and saturated NaCl solution (20 mL). The organic layer was dried over Na_2_SO_4_ and evaporated to dryness under reduced pressure. The residue was purified by flash chromatography on silica gel (eluent benzene—EtOAc from 95:5 to 9:1). Yield 1.16 g (88%). Colorless powder, m.p. 84.5–86.7 °C. TLC (CHCl_3_–EtOH 95:5): *R*_f_ 0.52. ^1^H NMR (DMSO-*d*_6_, 400 MHz) δ (ppm) (rotamers *I* and *II*, 85:15): −2.85 (br. s, 1H, BHB), −0.92–2.20 (br. m, 8H, 8 × BH), 0.94 (t, 12H, Bu_4_N^+^), 1.31 (sex, *J* = 7.4 Hz, 8H, Bu_4_N^+^), 1.53–1.61 (m, 8H, Bu_4_N^+^), 1.86 (s, 1.7H, 2 × CH-carborane, *I*), 2.23 (s, 0.3H, 2 × CH-carborane, *II*), 3.00–3.06 (m, 1.2H, Bu_4_N^+^, *II*), 3.14–3.18 (m, 6.8H, Bu_4_N^+^, *I*), 7.02 (br. s, 0.85H, NH, *I*), 7.18 (br. s, 0.15H, NH, *II*), 7.91 (s, 0.15H, CHO, *II*), 8.11 (d, *J* = 12.8 Hz, 0.85H, CHO, *I*). ^11^B NMR (DMSO-*d*_6_, 193 MHz) δ (ppm) (*major rotamer*): −37.8 (d, *J* = 132.4 Hz, 2B), −22.7 (d, *J* = 147.6 Hz, 2B), −17.3 (d, *J* = 132.2 Hz, 2B), −11.4 (d, *J* = 132.2 Hz, 2B), −8.97 (s, 1B). ^13^C NMR (DMSO-*d*_6_, 151 MHz) δ (ppm) (rotamers *I* and *II*): 14.0 (4C, Bu_4_N^+^), 19.7 (4C, Bu_4_N^+^), 23.5 (4C, Bu_4_N^+^), 45.3 (br. s, 2C, CH-carborane, *II*), 46.4 (br. s, 2C, CH-carborane, *I*), 58.0 (4C, Bu_4_N^+^), 164.5 (CHO, *II*), 166.7 (CHO, *I*). HRMS (ESI) (*m*/*z*) [M − Bu_4_N]^−^: calcd. for [C_3_H_13_^11^B_9_NO]^−^: 178.1846, found: 178.1845.

*3-Formamido-7,8-dicarba-nido-undecaborane Protonated Form* (**5b**). Amberlite IR-120 ion exchange resin (H form) (4 g) was added to a solution of compound **5a** (0.90 g, 2.15 mmol) in EtOH (70 mL). The mixture was stirred at room temperature for 1 h and filtered. A fresh portion of Amberlite IR-120 ion exchange resin (H form) (4 g) was added to the filtrate, and the mixture was stirred for 1 h and filtered. The filtrate was evaporated to dryness. Yield 0.38 g (100%). Yellowish hygroscopic foam. ^1^H NMR (DMSO-*d*_6_, 400 MHz) δ (ppm) (rotamers *I* and *II*, 85:15): −2.82 (br. s, 1H, BHB), −0.83–2.38 (br. m, 8H, 8 × BH), 1.86 (s, 1.7H, 2 × CH-carborane, *I*), 2.23 (s, 0.3H, 2 × CH-carborane, *II*), 4.63 (br. s, 1H, H^+^, overlapped by H_2_O signal), 7.04 (br. s, 0.85H, NH, *I*), 7.23 (br. s, 0.15H, NH, *II*), 7.91 (s, 0.15H, CHO, *II*), 8.11 (d, *J* = 12.5 Hz, 0.85H, CHO, *I*). ^11^B NMR (DMSO-*d*_6_, 193 MHz) δ (ppm) (*major rotamer*): −37.8 (d, *J* = 132.6 Hz, 2B), −22.8 (d, *J* = 147.2 Hz, 2B), −17.3 (d, *J* = 130.2 Hz, 2B), −11.4 (d, *J* = 135.9 Hz, 2B), −9.0 (s, 1B). ^13^C NMR (DMSO-*d*_6_, 151 MHz) δ (ppm) (rotamers *I* and *II*): 45.2 (br. s, 2C, CH-carborane, *II*), 46.4 (br. s, 2C, CH-carborane, *I*), 164.9 (CHO, *II*), 166.9 (CHO, *I*). HRMS (ESI) (*m*/*z*) [M − H]^−^: calcd. for [C_3_H_13_^11^B_9_NO]^−^: 178.1846, found: 178.1844.

*3-Formamido-7,8-dicarba-nido-undecaborane Potassium Salt, Solvate with EtOH* (**5c** × 0.5EtOH). Compound **5b** (0.38 g, 2.14 mmol) was added to a solution of KOH (0.18 g, 3.21 mmol) in EtOH (20 mL). The reaction mixture was stirred at room temperature for 30 min, then solid CO_2_ (excess) was added, and the precipitate was filtered off. The filtrate was evaporated to dryness under reduced pressure. Yield 0.51 g (100%). Colorless hygroscopic foam. ^1^H NMR (DMSO-*d*_6_, 400 MHz) δ (ppm) (rotamers *I* and *II*, 85:15): −2.87 (br. s, 1H, BHB), −0.80–2.45 (br. m, 8H, 8 × BH), 1.86 (s, 1.7H, 2 × CH-carborane, *I*), 2.24 (s, 0.3H, 2 × CH-carborane, *II*), 7.03 (br. s, 0.85H, NH, *I*), 7.19 (br. s, 0.15H, NH, *II*), 7.91 (s, 0.15H, CHO, *II*), 8.11 (d, *J* = 12.5 Hz, 0.85H, CHO, *I*). ^11^B NMR (DMSO-*d*_6_, 193 MHz) δ (ppm) (*major rotamer*): −37.8 (d, *J* = 133.8 Hz, 2B), −22.8 (d, *J* = 148.1 Hz, 2B), −17.3 (d, *J* = 131.2 Hz, 2B), −11.4 (d, *J* = 132.5 Hz, 2B), −9.0 (s, 1B). ^13^C NMR (DMSO-*d*_6_, 151 MHz) δ (ppm) (rotamers *I* and *II*): 45.3 (br. s, 2C, CH-carborane, *II*), 46.5 (br. s, 2C, CH-carborane, *I*), 164.6 (CHO, *II*), 166.8 (CHO, *I*). HRMS (ESI) (*m*/*z*) [M − K]^−^: calcd. for [C_3_H_13_^11^B_9_NO]^−^: 178.1846, found: 178.1846.

*3-Amino-9-dimethylsulfonio-7,8-dicarba-nido-undecaborane* (**2**).

*Method A.* Compound **2′** (0.10 g, 0.33 mmol) was added to 5% NaHCO_3_ solution (7 mL) with stirring at room temperature, then EtOAc (5 mL) was added. The aqueous layer was separated and washed with EtOAc (3 × 5 mL). The combined organic layers were washed with water (7 mL) and saturated NaCl solution (7 mL), dried with Na_2_SO_4_, and evaporated to dryness under reduced pressure. Yield 0.07 g (96%). Colorless powder, m.p. 110–114 °C. TLC (CHCl_3_–EtOH 4:6): *R*_f_ 0.24. ^1^H NMR (CDCl_3_, 500 MHz) δ (ppm): −3.09 (br. s, 1H, BHB), 0.1–2.4 (br. m, 7H, 7 × BH), 1.07 (s, 2H, NH_2_), 2.27 (s, 1H, CH-carborane), 2.59 (s, 3H, Me), 2.66 (s, 1H, CH-carborane), 2.75 (s, 3H, Me). ^1^H{^11^B} NMR (CDCl_3_, 500 MHz) δ (ppm): −3.09 (s, 1H, H-*ec*), 0.51 (s, 1H, H-10), 0.90 (s, 1H, H-6),1.07 (s, 3H, H-1, NH_2_), 1.20 (s, 1H, H-4), 2.12 (s, 1H, H-2), 2.28 (br. s, 2H, H-5, H-7), 2.58 (s, 3H, Me), 2.65 (s, 1H, H-8), 2.75 (s, 3H. Me). ^11^B NMR (CDCl_3_, 160 MHz) δ (ppm): −36.4 (d, *J* =139.5 Hz, 1B, B-10), −35.8 (d, *J* =140.5 Hz, 1B, B-1), −27.1 (d, *J* = 144.7 Hz, 1B, B-6), −24.1 (d, *J* = 150.9 Hz, 1B, B-4), −17.8 (d, *J* = 140.6 Hz, 1B, B-11), −12.6 (d, *J* = 153.9 Hz, 1B, B-2), −8.0 (s, 1B, B-9), −5.2 (d, *J* = 111.1 Hz, 1B, B-5), −4.9 (s, 1B, B-3). ^13^C NMR (CDCl_3_, 126 MHz) δ (ppm): 25.5 (Me), 28.6 (Me), 45.8 (br. s, C-7), 58.4 (br. s, C-8). HRMS (ESI) (*m*/*z*) [M + H]^+^: calcd. for [C_4_H_19_^11^B_9_NS]^+^: 212.2079, found: 212.2080.

*Method B.* Water (2 mL) and H_2_SO_4_ conc. (2.53 mL) were slowly added to a solution of compound **5c** × 0.5EtOH (0.42 g, 1.76 mmol) in DMSO (0.55 mL) under argon atmosphere under stirring at room temperature. The reaction mixture was stirred at 80 °C for 4.5 h, then cooled to room temperature. Solid KOH was added to the reaction mixture to pH ~ 8, then the reaction mixture was extracted with EtOAc (4 × 15 mL). The combined organic layers were washed with water (10 mL), saturated NaCl solution (10 mL), dried with Na_2_SO_4_, and evaporated to dryness under reduced pressure. The residue was purified by flash chromatography on silica gel (eluent CHCl_3_–EtOH from 8:2 to 2:8). Yield 0.25 g (68%). Colorless amorphous solid. TLC (CHCl_3_–EtOH 4:6): *R*_f_ 0.24. ^1^H, ^11^B, and ^13^C NMR spectra were identical to those of compound **2** obtained according to *method A*. HRMS (ESI) (*m*/*z*) [M + H]^+^: calcd. for [C_4_H_19_^11^B_9_NS]^+^: 212.2079, found: 212.2081.

*3-Ammonio-9-methylthio-7,8-dicarba-nido-undecaborane* (**6**). Sodium (0.50 g, 21.92 mmol) was added to a solution of naphthalene (3.09 g, 24.11 mmol) in THF (26 mL) under stirring at 10 °C. The reaction mixture was stirred at 10 °C for 15 min, then a solution of compound 2 (1.70 g, 8.12 mmol) in THF (6 mL) was added dropwise under stirring. The reaction mixture was stirred at room temperature for 7 days and cooled to 10 °C. MeOH (14 mL) was added to the reaction mixture. The reaction mixture was evaporated to dryness under reduced pressure. The residue was dissolved in water (30 mL), and solid NaOH was added to pH = 14. The reaction mixture was extracted with *n*-hexane (3 × 15 mL). The combined organic layers were washed with 2N NaOH (2 × 5 mL). The combined aqueous layers were acidified with HCl conc. to pH ~ 2 and extracted with EtOAc (4 × 15 mL). The combined organic layers were washed with saturated aqueous NaCl solution (2 × 10 mL), dried over Na_2_SO_4,_ and evaporated to dryness under reduced pressure. The residue was purified by flash chromatography on silica gel (eluent CHCl_3_–EtOH from 95:5 to 8:2). Yield 1.21 g (76%). Beige powder, m.p. 271–277 °C (decomp.). TLC (CHCl_3_–EtOH 9:1): *R*_f_ 0.30. HPLC (Chiralpak AS-H, *n*-hexane–*i*PrOH 5:1, 1.0 mL/min, detection at 220 nm): *τ*_1_ 8.68 min, *τ*_2_ 12.75 min. ^1^H NMR (DMSO-*d*_6_, 500 MHz) δ (ppm): −2.50 (br. s, 1H, BHB), −0.4–2.5 (br. m, 7H, 7×BH), 1.93 (s, 1H, CH-carborane), 1.95 (s, 3H, Me), 2.34 (s, 1H, CH-carborane), 7.23 (br. s, 3H, NH_3_^+^). ^11^B{^1^H} NMR (DMSO-*d*_6_, 128 MHz) δ (ppm): −38.6 (1B), −36.1 (1B), −23.3 (3B), −11.9 (3B), 1.8 (1B). ^13^C NMR (DMSO-*d*_6_, 126 MHz) δ (ppm): 15.6 (Me), 37.9 (CH-carborane), 48.7 (br. s, CH-carborane). HRMS (ESI) (*m*/*z*) [M − H]^−^: calcd. for [C_3_H_15_^11^B_9_NS]^−^: 196.1776, found: 196.1776.

*Alkylation of Compound **6** with tert-Butyl Iodoacetate*. A solution of compound **6** (0.26 g, 1.32 mmol) and *tert*-butyl iodoacetate (0.32 g, 1.32 mmol) in EtOH (15 mL) was stirred under reflux for 10 h, then cooled to room temperature and evaporated to dryness under reduced pressure. The residue was separated by flash chromatography on silica gel (CHCl_3_ to CHCl_3_–EtOH 7:3) to afford amino ester **7** (in the form of hydroiodide) and compound **2”** as fast and slow eluting components, respectively.

*tert-Butyl 2-[S-(3-Ammonio-7,8-dicarba-nido-undecaboran-9-yl)-S-methylsulfonio]ethanoate Iodide* (**7**). Yield 0.32 g (55%). Yellowish powder, m.p. 77–79 °C. TLC (CHCl_3_–EtOH 9: 1): *R*_f_ 0.48. ^1^H NMR (DMSO-*d*_6_, 500 MHz) δ (ppm) (diastereomers *I* and *II*, appr. 6:4): −3.11 (br. s, 1H, BHB (*I* and *II*)), −0.2–2.5 (br. m, 7H, 7 × BH), 1.46 (s, 3.6H, *t*Bu (*II*)), 1.49 (s, 5.4H, *t*Bu (*I*)), 2.38–2.45 (br. s, 1H, CH-carborane (*I* and *II*)), 2.70 (s, 1.8H, SMe (*I*)), 2.84 (s, 1.2H, SMe (*II*)), 3.07–3.12 (br. s, 1H, CH-carborane (*I* and *II*)), 4.04 (d, *J* = 15.8 Hz, 0.4H, H_B_ (*II*)), 4.20 (d, *J* = 15.6 Hz, 0.6H, H_B_ (*I*)), 4.37 (d, *J* = 15.8 Hz, 0.4H, H_A_ (*II*)), 4.50 (d, *J* = 15.6 Hz, 0.6H, H_A_ (*I*)), 7.49 (br. s, 3H, NH_3_^+^). ^11^B{^1^H} NMR (DMSO-*d*_6_, 160 MHz) δ (ppm): −37.6 (br. s, 1B), −33.2 (br. s, 1B), −26.7 (1B), −24.1 (1B), −12.4 (2B), −5.1 (br. s, 3B). ^13^C NMR (DMSO-*d*_6_, 126 MHz) δ (ppm) (diastereomers *I* and *II*): 22.8 (SMe (*I*)), 25.2 (SMe (*II*)), 27.49 (*t*Bu (*II*)), 27.52 (*t*Bu (*I*)), 37.9 (br. s, carborane (*I* and *II*)), 44.4 (CH_2_ (*II*)), 46.7 (CH_2_ (*I*)), 52.6 (br. s, CH-carborane (*II*)), 53.6 (br. s, CH-carborane (*I*)), 83.6 (*t*Bu (*II*)), 83.8 (*t*Bu (*I*)), 164.0 (CO (*II*)), 164.4 (CO (*I*)). HRMS (ESI) (*m*/*z*) [M − I]^+^: calcd. for [C_9_H_27_^11^B_9_NO_2_S]^+^: 312.2611, found: 312.2609. HRMS (APCI) (*m*/*z*) [I]^−^ calcd.: 126.9050, found 126.9050.

*3-Ammonio-9-dimethylsulfonio-7,8-dicarba-nido-undecaborane Iodide* (**2″**). Yield 0.045 g (10%). Beige amorphous powder. TLC (CHCl_3_–EtOH 9:1): *R*_f_ 0.19. ^1^H NMR (DMSO-*d*_6_, 500 MHz) δ (ppm): −3.09 (br. s, 1H, BHB), 0.1–2.5 (br. m, 7H, 7 × BH), 2.42 (s, 1H, CH-carborane), 2.67 (s, 3H, Me), 2.81 (s, 3H, Me), 3.10 (s, 1H, CH-carborane), 7.40 (br. s, 3H, NH_3_). ^11^B NMR (DMSO-*d*_6_, 160 MHz) δ (ppm): −37.7 (d, *J* = 140.2 Hz, 1B), −33.9 (br. s, 1B), −26.8 (d, *J* = 141.2 Hz, 1B), −24.1 (d, *J* = 136.8 Hz, 1B), −12.1 (br. s, 2B), −5.2 (br. s, 3B). ^13^C NMR (DMSO-*d*_6_, 126 MHz) δ (ppm): 24.7 (Me), 26.75 (Me), 39.5 (CH-carborane, overlapped by DMSO-*d*_6_ signal), 53.0 (br. s, CH-carborane). HRMS (ESI) (*m*/*z*) [M − I]^+^: calcd. for [C_4_H_19_^11^B_9_NS]^+^: 212.2079, found: 212.2079.

*2-[S-(3-Amino-7,8-dicarba-nido-undecaboran-9-yl)-S-methylsulfonio]ethanoic Acid* (**1**). Trifluoroacetic acid (1.9 mL) was added to a solution of compound **7** (0.19 g, 0.44 mmol) in CH_2_Cl_2_ (2.6 mL). The reaction mixture was stirred at room temperature for 3 h, then evaporated to dryness under reduced pressure. The residue was treated with Et_2_O (15 mL). The precipitate was filtered off and then purified by flash chromatography on silica gel (CHCl_3_–EtOH from 7:3 to 1:1). Yield 0.08 g (72%). Colorless powder, m.p. 252–255 °C. TLC (CHCl_3_–EtOH 1:1): *R*_f_ 0.39. ^1^H NMR (DMSO-*d*_6_, 500 MHz) δ (ppm) (diastereomers *I* and *II*, appr. 7:3): −3.14 (br. s, 1H, BHB (*I* and *II*)), −0.1–2.5 (br. m, 7H, 7×BH), 2.46 (s, 2.1H, SMe (*I*)), 2.48 (br. s, 1H, CH-carborane (*I* and *II*)), 2.66 (s, 0.9H, SMe (*II*)), 3.20 (br. s, 0.3H, CH-carborane (*II*)), 3.31 (br. s, 0.7H, CH-carborane (*I*)), 3.57 (d, *J* = 15.0 Hz, 0.3H, H-2B (*II*)), 3.63 (d, *J* = 14.5 Hz, 0.7H, H-2B (*I*)), 3.68 (d, *J* = 15.0 Hz, 0.3H, H-2A (*II*)), 4.05 (d, *J* = 14.5 Hz, 0.7H, H-2A (*I*)), 7.93 (br. s, 3H, NH_3_^+^). ^11^B{^1^H} NMR (DMSO-*d*_6_, 160 MHz) δ (ppm): −37.7 (1B), −34.2 (br. s, 1B), −27.0 (1B), −24.0 (br. s, 1B), −14.5 (br. s, 2B), −12.0 (1B), −4.9 (br. s, 2B). ^13^C NMR (DMSO-*d*_6_, 126 MHz) δ (ppm) (diastereomers *I* and *II*): 21.7 (SMe (*I*)), 24.2 (SMe (*II*)), 39.5 (CH-carborane (*I* and *II*), overlapped by DMSO-*d*_6_ signal), 47.6 (CH_2_ (*II*)), 51.4 (CH_2_ (*I*)), 52.7 (br. s, CH-carborane (*II*)), 54.4 (br. s, CH-carborane (*I*)), 165.2 (CO_2_H (*II*)), 166.2 (CO_2_H (*I*)). HRMS (ESI) (*m*/*z*) [M + H]^+^: calcd. for [C_5_H_19_^11^B_9_NO_2_S]^+^: 256.1980, found: 256.1978.

*9-Dimethylsulfonio-7,8-dicarba-nido-undecaborane* (**9**). A solution of compound **7** (17.8 mg, 0.041 mmol) and compound **8** (12.7 mg, 0.041 mmol) in EtOH (1 mL) was stirred under reflux for 28 h, then evaporated to dryness under reduced pressure. The residue was purified by flash chromatography (*n*-hexane–EtOAc from 8:2 to 4:6). Yield 3.4 mg (43%). Beige powder, m.p. 138–141 °C (lit. m.p. 147–148 °C [110]). TLC (*n*-hexane–EtOAc 6:4): *R*_f_ 0.34. HRMS (ESI) (*m*/*z*) [M + Na]^+^: calcd. for [C_4_H_17_^11^B_9_NaS]^+^: 219.1790, found: 219.1788. ^1^H NMR spectrum was identical to that described in [87].

## 4. Conclusions

Thus, we have developed a method for the preparation of a new *nido*-carborane-based bifunctional building block, namely 3-ammonio-9-methylthio-7,8-dicarba-*nido*-undecaborane. Its alkylation with *tert*-butyl iodoacetate yielded a charge-compensated *nido*-carborane-based amino ester as a mixture of diastereomers in a ratio of approximately 6:4. Removal of the ester protecting group led to a new planar-chiral amino acid with free functional groups. In its molecule, the amino group is directly attached to the B(3) atom, and the carboxyl group is linked to the B(9) atom via the CH_2_S^+^(Me) fragment. For the first time, the assignment of boron atom signals in the ^11^B NMR spectrum of the 3,9-disubstituted *nido*-carborane derivative was carried out. The obtained *nido*-carborane-based planar-chiral amino acid, containing a positively charged sulfur atom, and related compounds are of interest as a basis for peptide-like compounds and chiral ligands.

## Data Availability

The original contributions presented in the study are included in the article/Supplementary Material, further inquiries can be directed to the corresponding author.

## Supplementary Materials

The following supporting information can be downloaded at: https://www.mdpi.com/article/10.3390/molecules29184487/s1, Figures S1–S32: ^1^H, ^11^B, and ^13^C NMR spectra of compounds **2′**, **5a**–**c**, **2**, **6**, **7**, **2″** and **1**; Figure S33: HPLC chromatogram of compound **6**; Figures S34–S43: HRMS data for compounds **2′**, **5a**–**c**, **2**, **6**, **7**, **2″**, **1** and **9**.
