# Supplementary material for: Synthesis of Novel Planar-Chiral Charge-Compensated nido-Carborane-Based Amino Acid
Source: Molecules. 2024 Sep 21;29(18):4487. doi: 10.3390/molecules29184487 (PMC11434195; doi:10.3390/molecules29184487)
Supplement: Supplementary file 1 [file molecules-29-04487-s001.zip › molecules-3206882-supplementary.pdf]

Supplementary Materials

Synthesis of Novel Planar-Chiral Charge-Compensated *nido*-Carborane-Based Amino Acid

Dmitry A. Gruzdev [1,*], Angelina A. Telegina [1], Marina A. Ezhikova [1], Mikhail I. Kodess [1], Galina L. Levit [1] and Victor P. Krasnov [1]

[1] Postovsky Institute of Organic Synthesis, Russian Academy of Sciences (Ural Branch), Ekaterinburg 620108, Russia

Correspondence: gruzdev-da@ios.uran.ru (D.A.G.)

Table of Content

NMR Spectra S-2

HPLC Data S-18

HRMS Data S-19

NMR Spectra

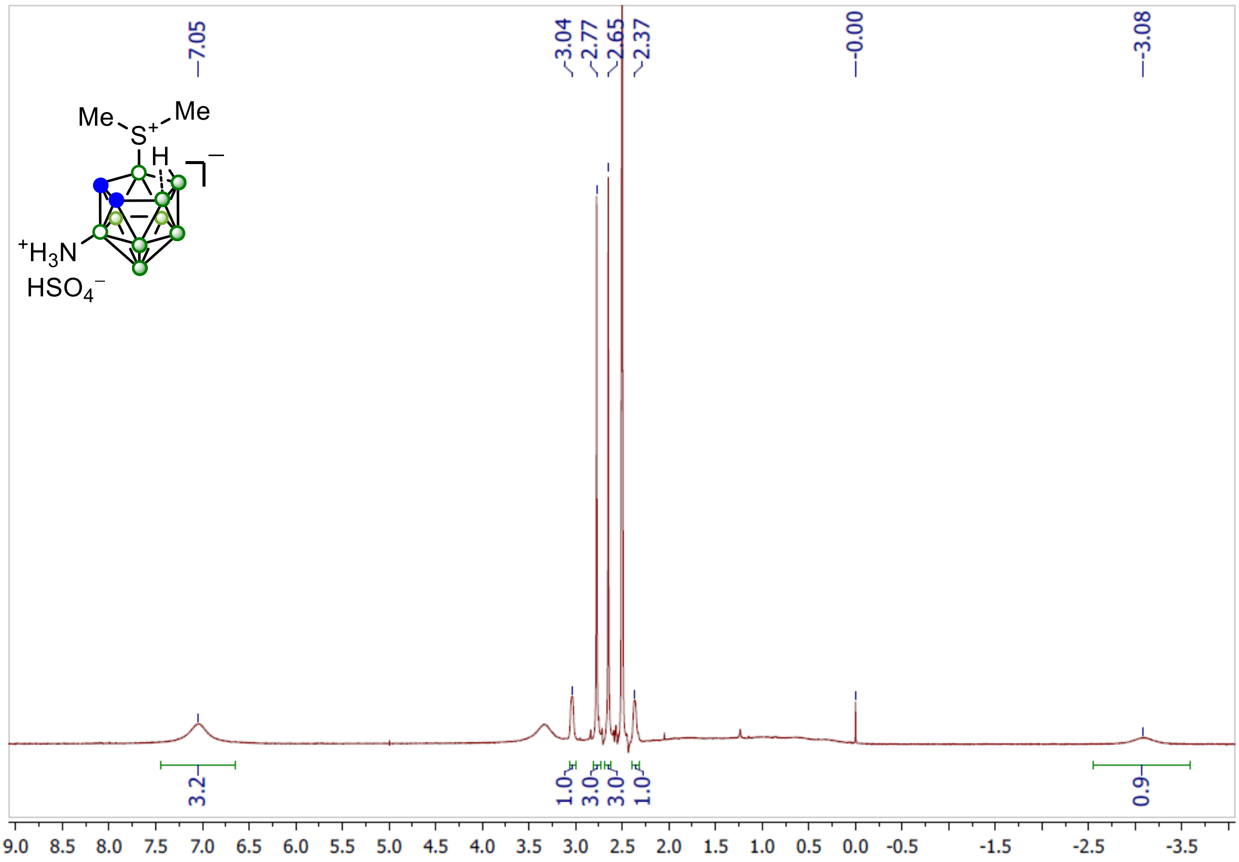

Figure S1. ^{1}H NMR spectrum of compound **2’** (DMSO-d_6, 400 MHz)

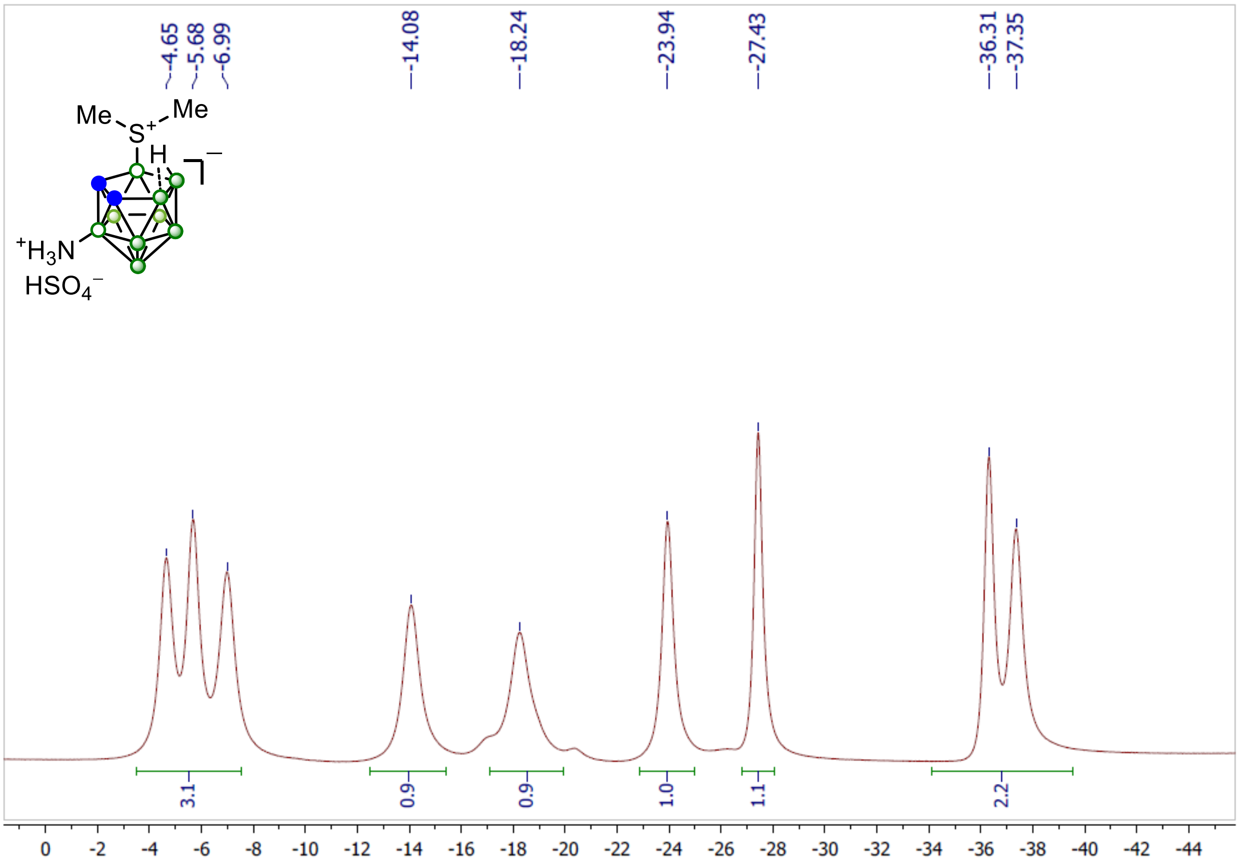

Figure S2. $^{11}B\{^{1}H\}$ NMR spectrum of compound **2’** (DMSO-d_6, 193 MHz)

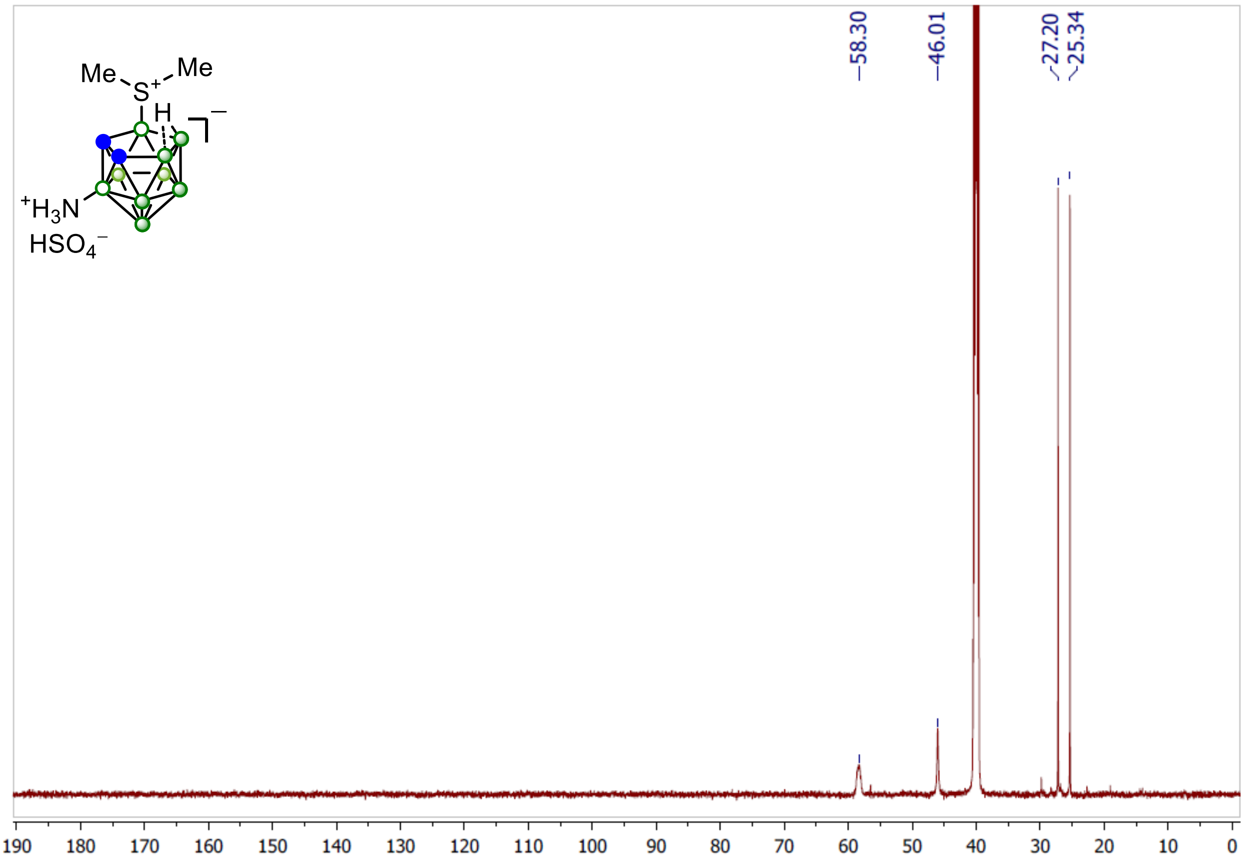

Figure S3. ^{13}C NMR spectrum of compound **2’** (DMSO-d_6, 151 MHz)

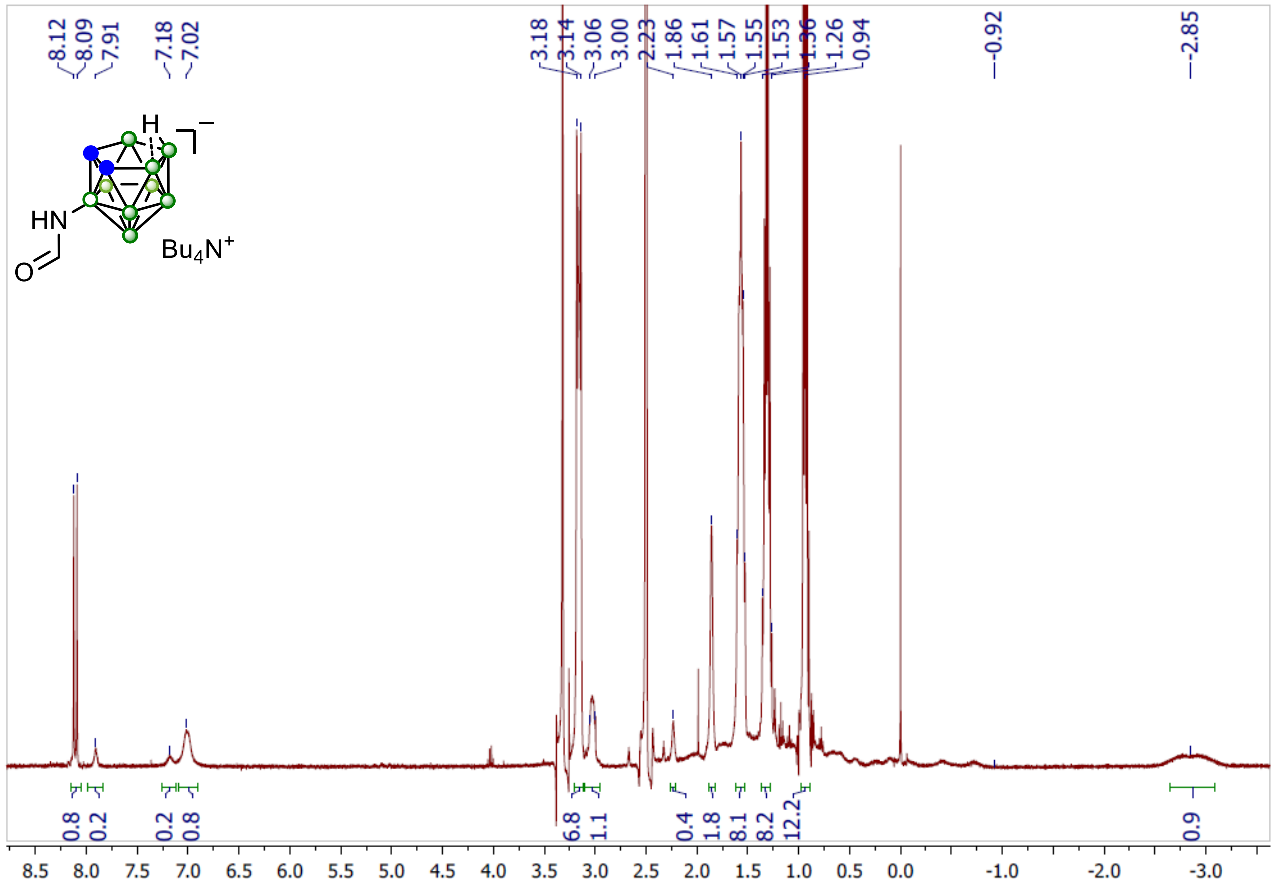

Figure S4. ^{1}H NMR spectrum of compound **5a** (DMSO-d_6, 400 MHz)

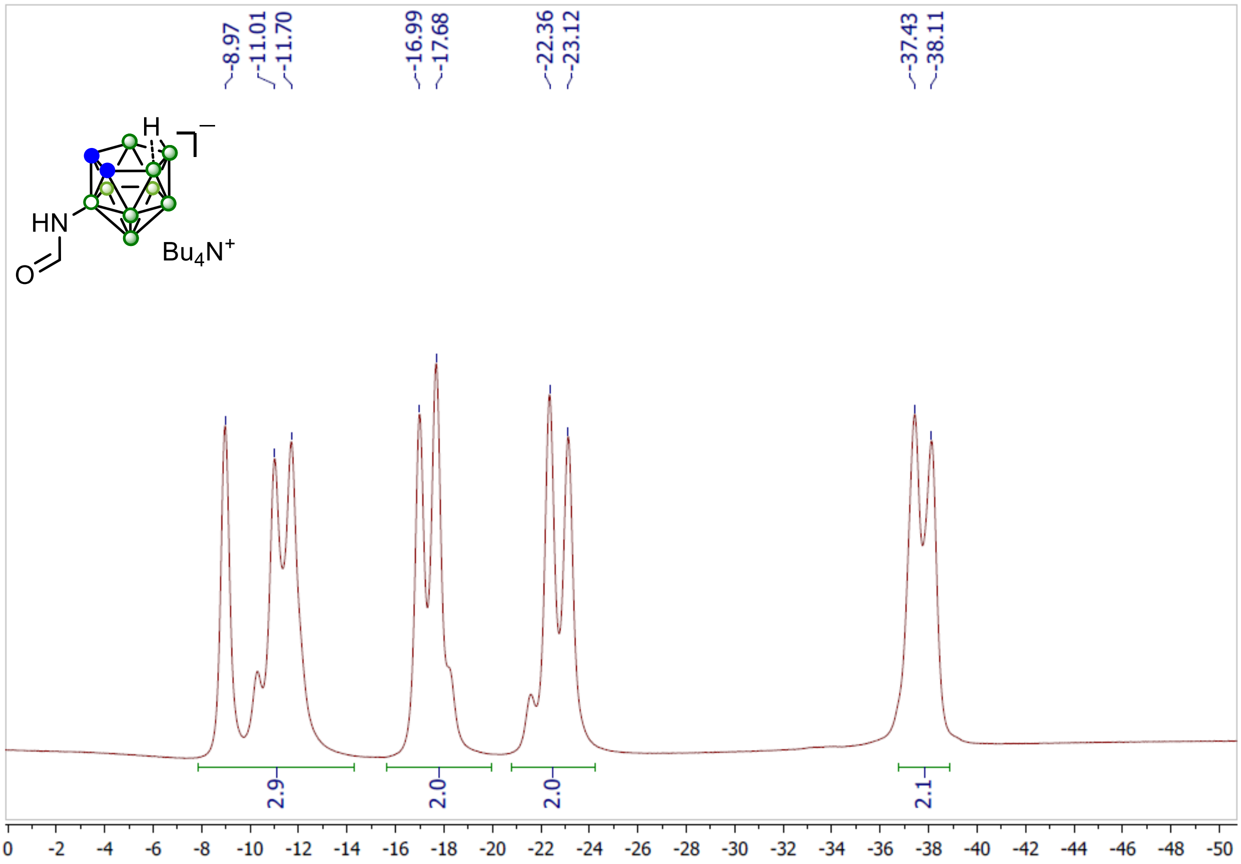

Figure S5. ^{11}B NMR spectrum of compound **5a** (DMSO-d_6, 193 MHz)

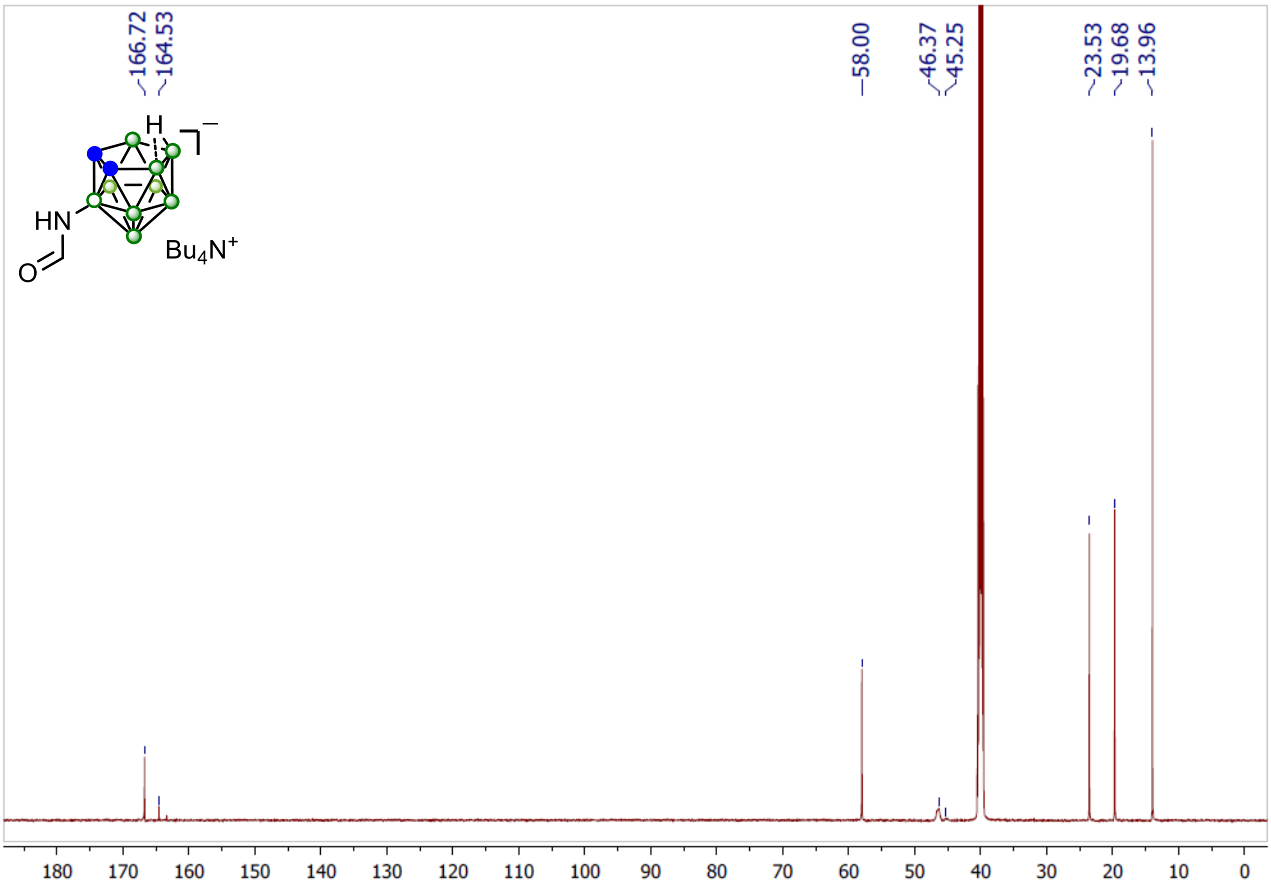

Figure S6. ^{13}C NMR spectrum of compound **5a** (DMSO-d_6, 151 MHz)

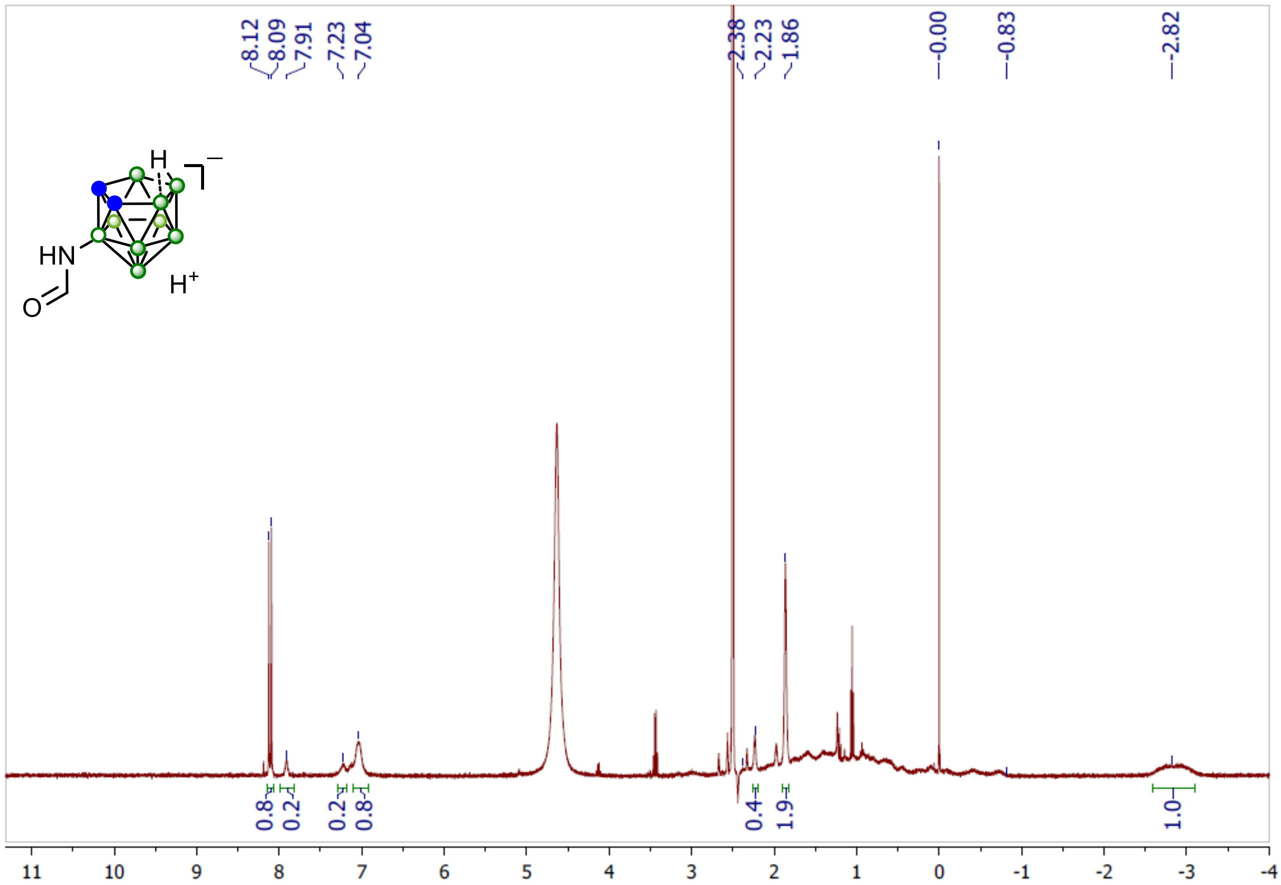

Figure S7. ^{1}H NMR spectrum of compound **5b** (DMSO-d_6, 400 MHz)

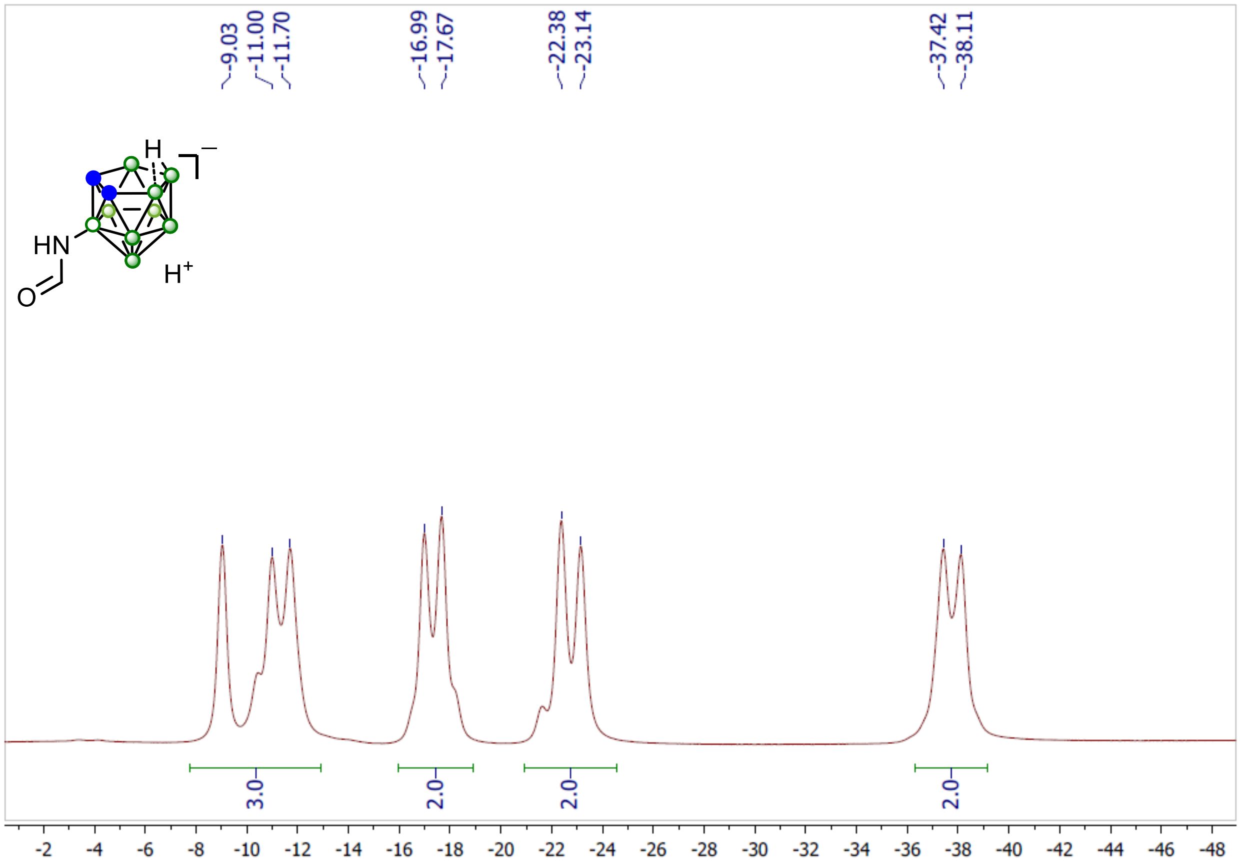

Figure S8. ^{11}B NMR spectrum of compound **5b** (DMSO-d_6, 193 MHz)

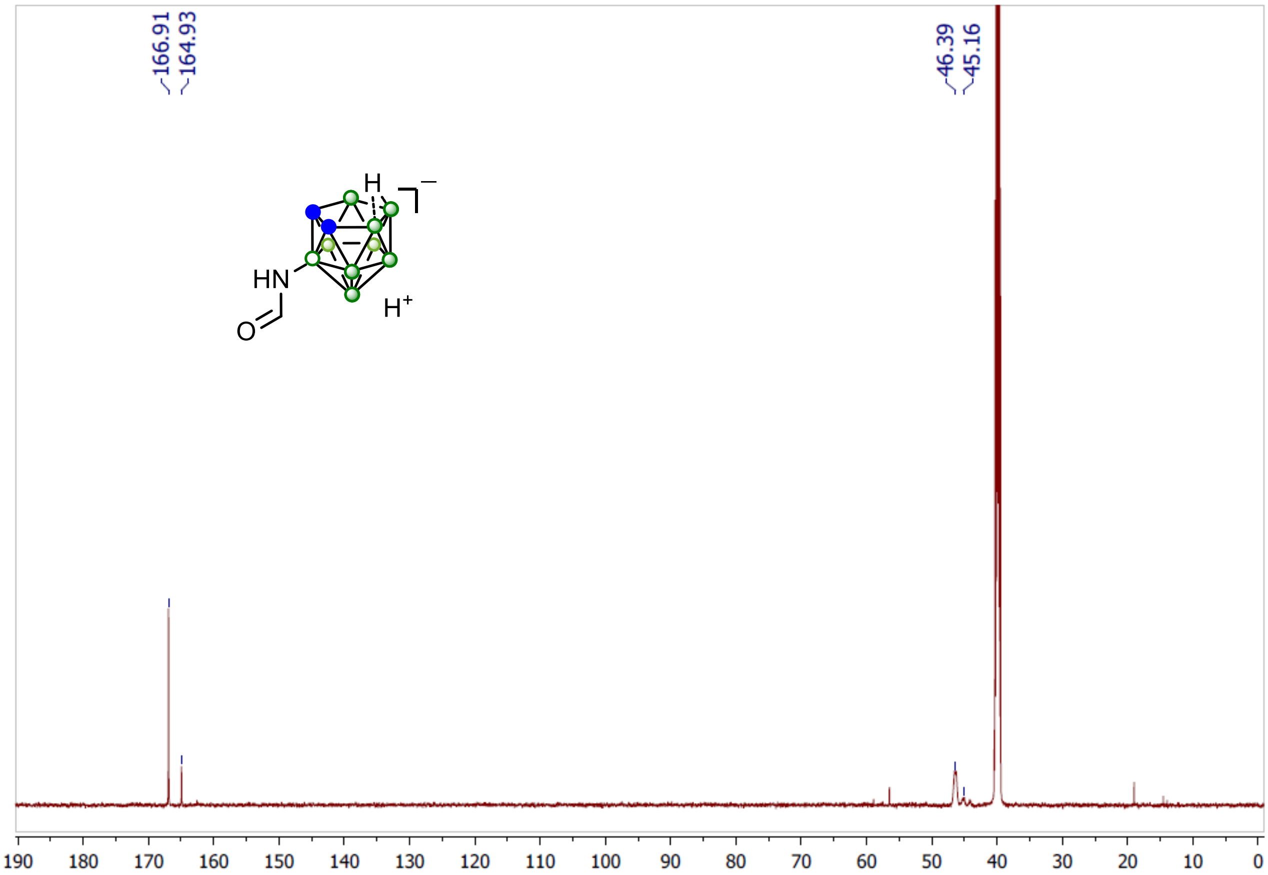

Figure S9. ^{13}C NMR spectrum of compound **5b** (DMSO-d_6, 151 MHz)

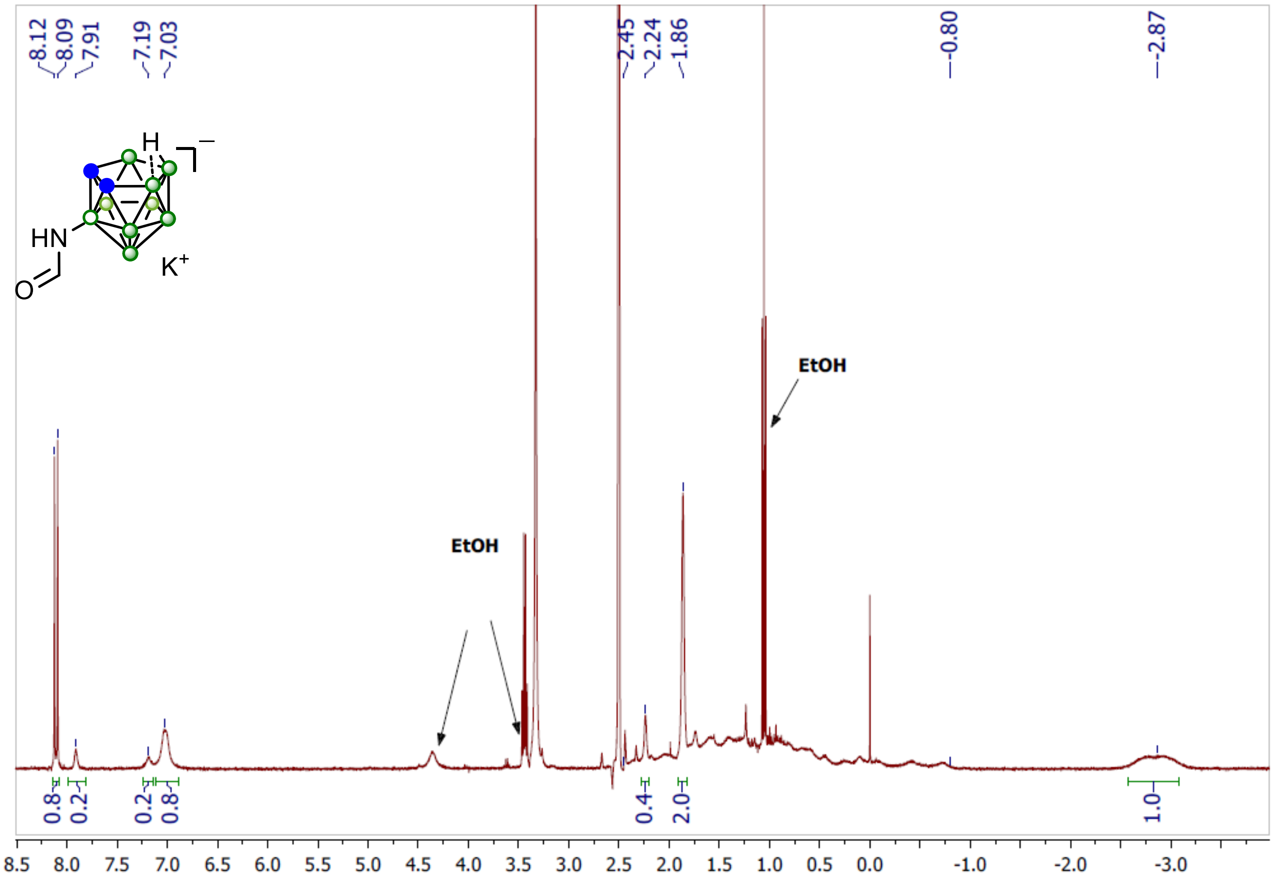

Figure S10. ^{1}H NMR spectrum of compound **5c** (DMSO-d_6, 400 MHz)

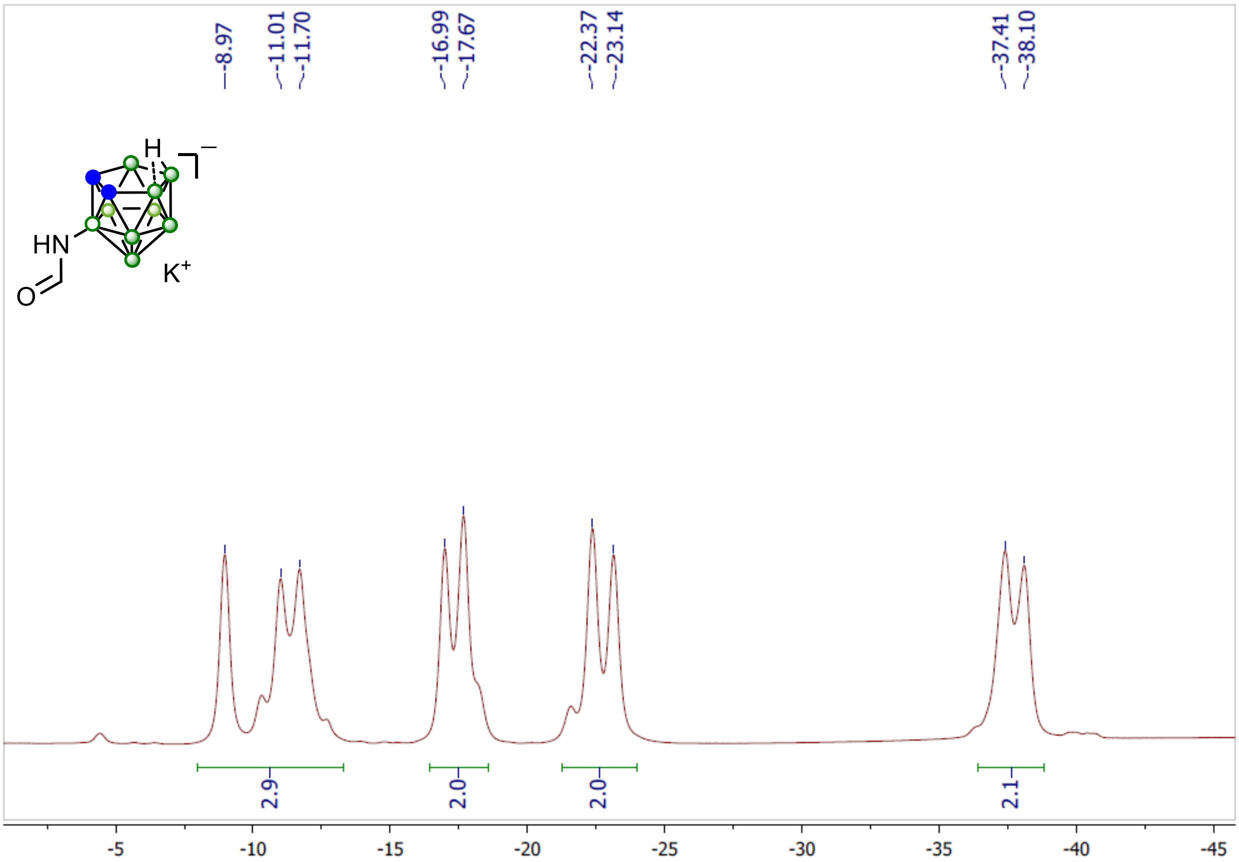

Figure S11. ^{11}B NMR spectrum of compound **5c** (DMSO-d_6, 193 MHz)

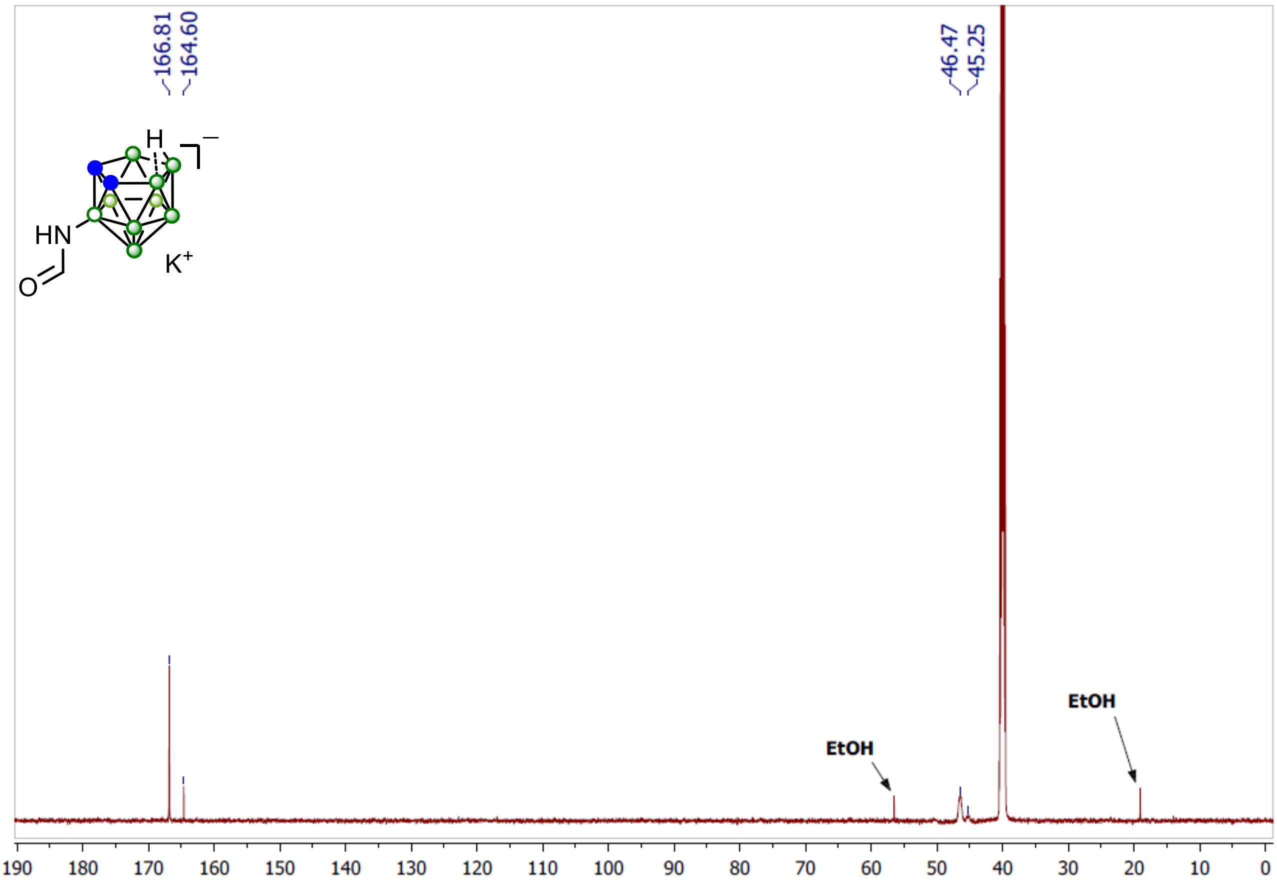

Figure S12. ^{13}C NMR spectrum of compound **5c** (DMSO-d_6, 151 MHz)

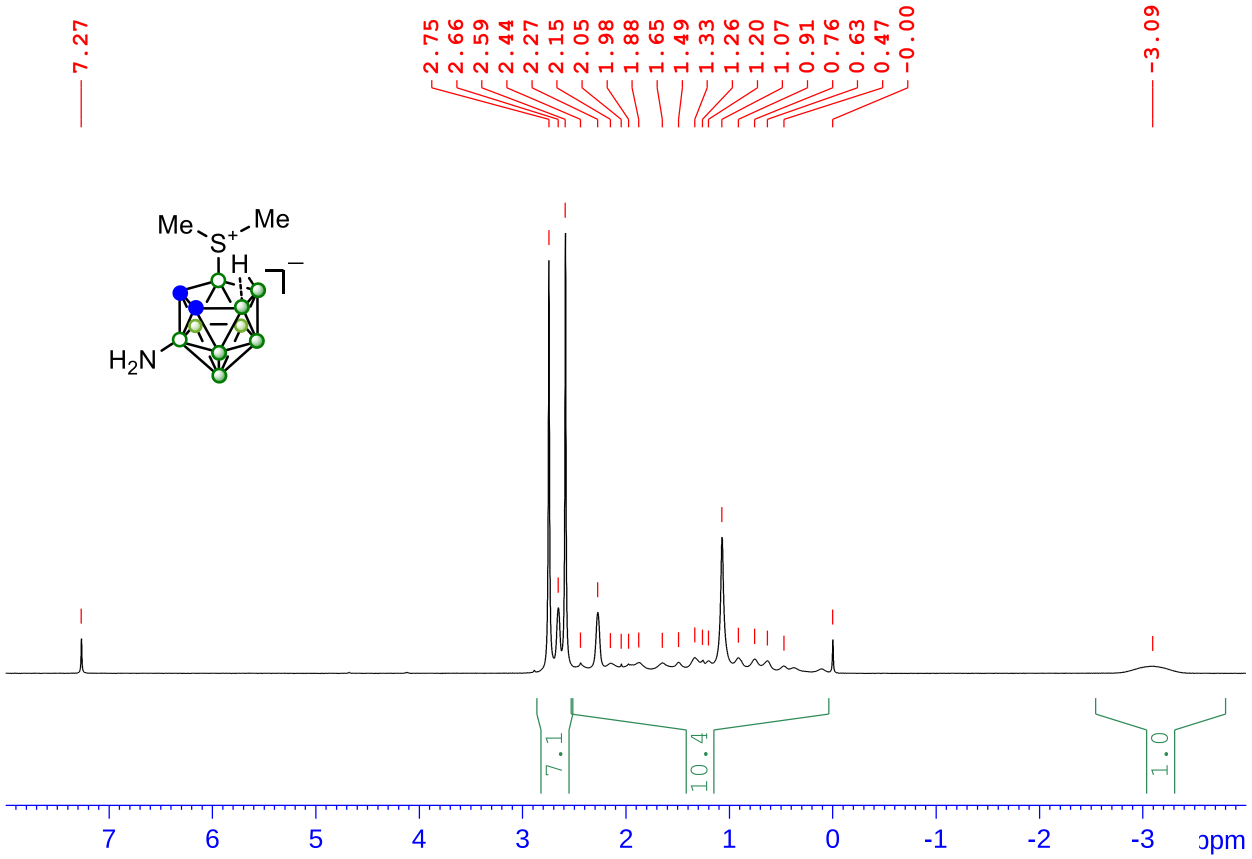

Figure S13. ^{1}H NMR spectrum of compound **2** ($CDCl_3$, 500 MHz)

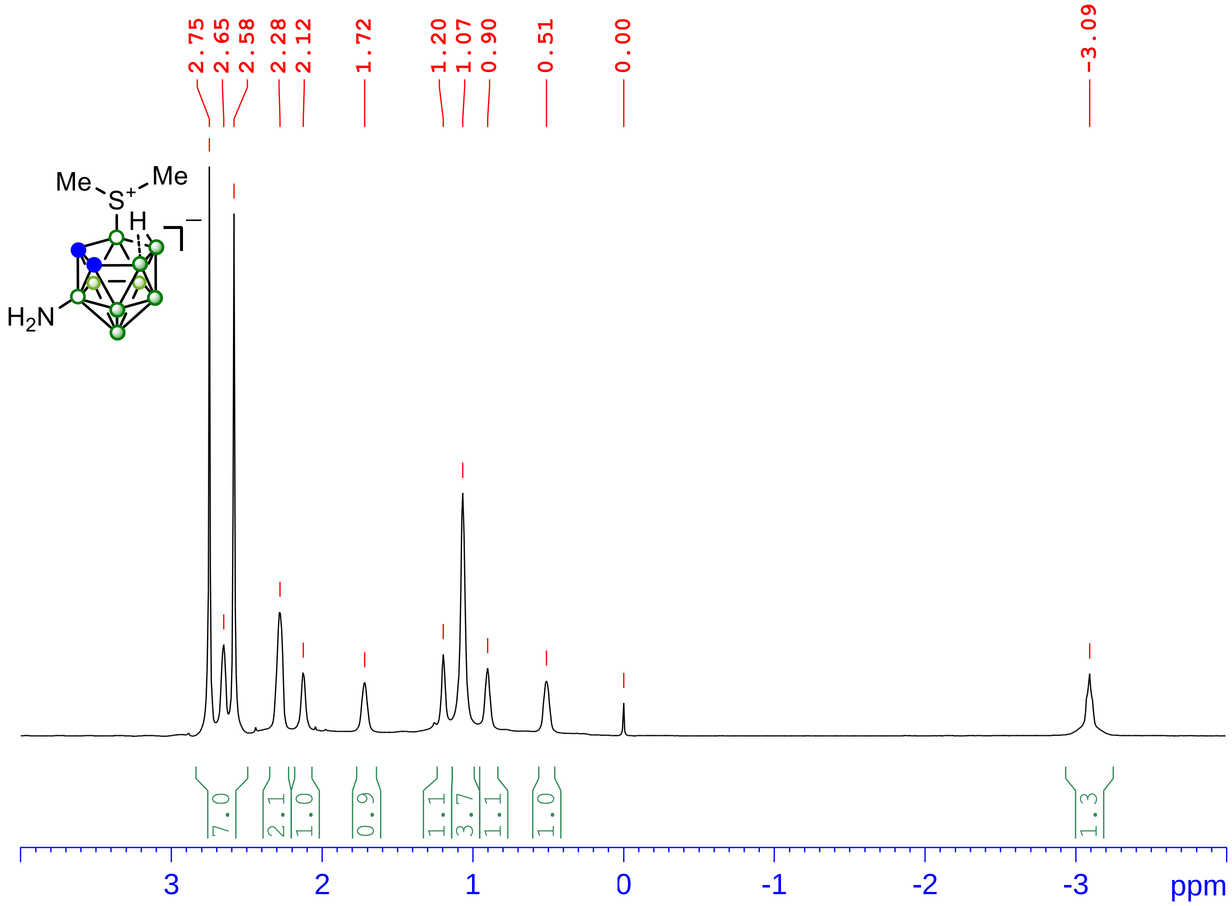

Figure S14. $^{1}H\{^{11}B\}$ NMR spectrum of compound **2** ($CDCl_3$, 500 MHz)

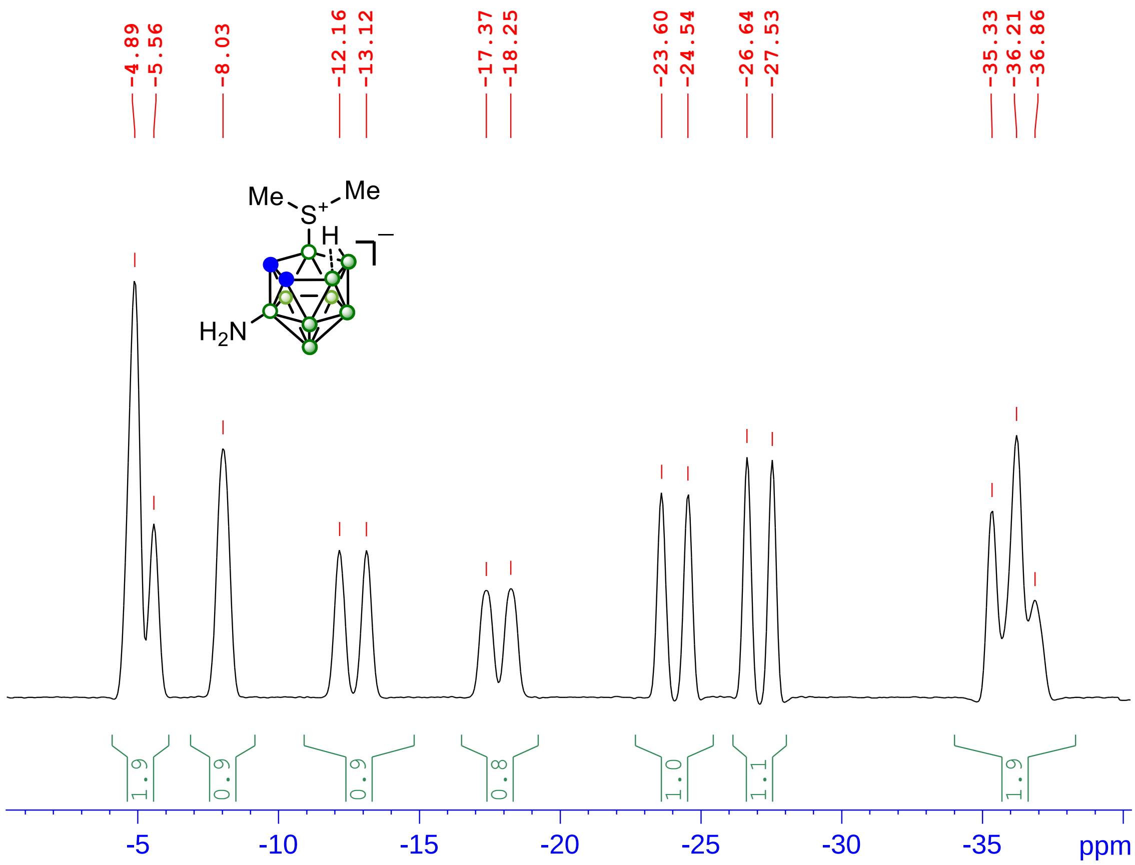

Figure S15. ^{11}B NMR spectrum of compound **2** ($CDCl_3$, 160 MHz)

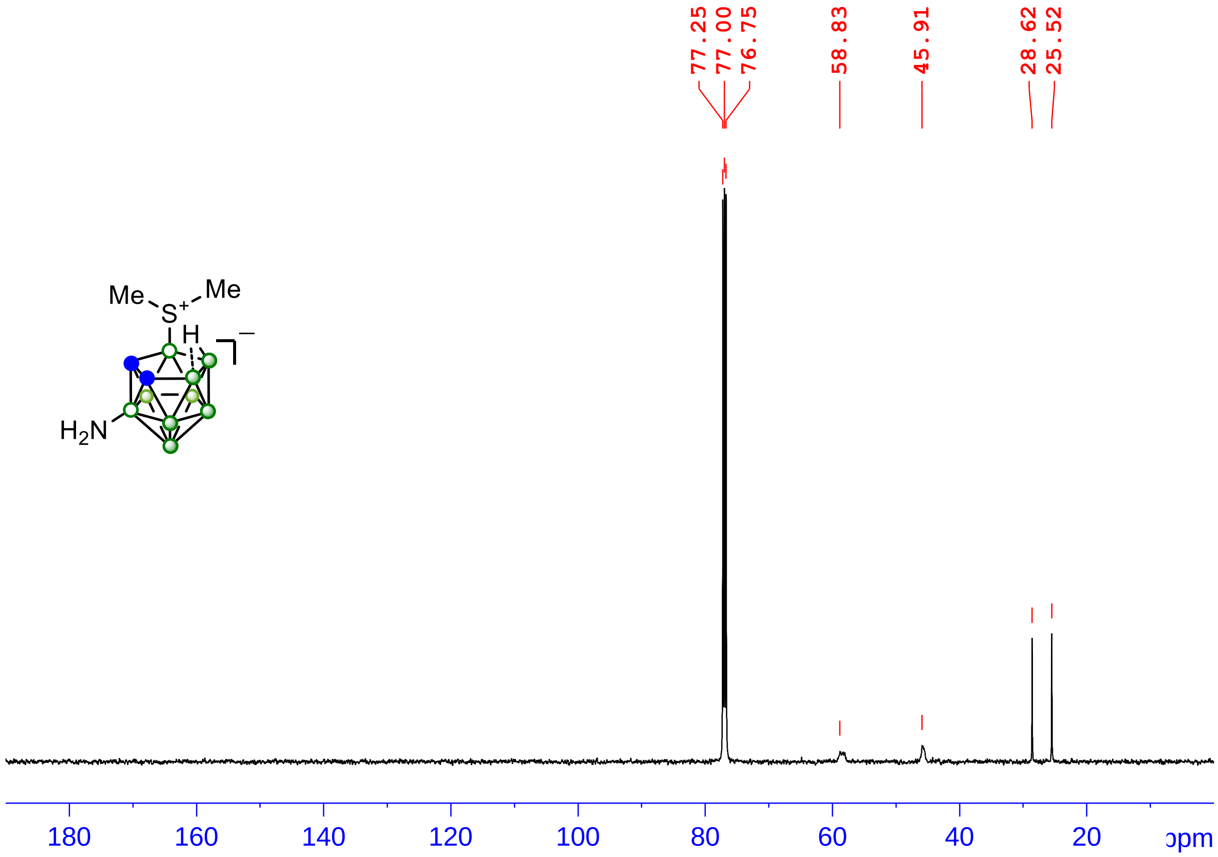

Figure S16. ^{13}C NMR spectrum of compound **2** ($CDCl_3$, 126 MHz)

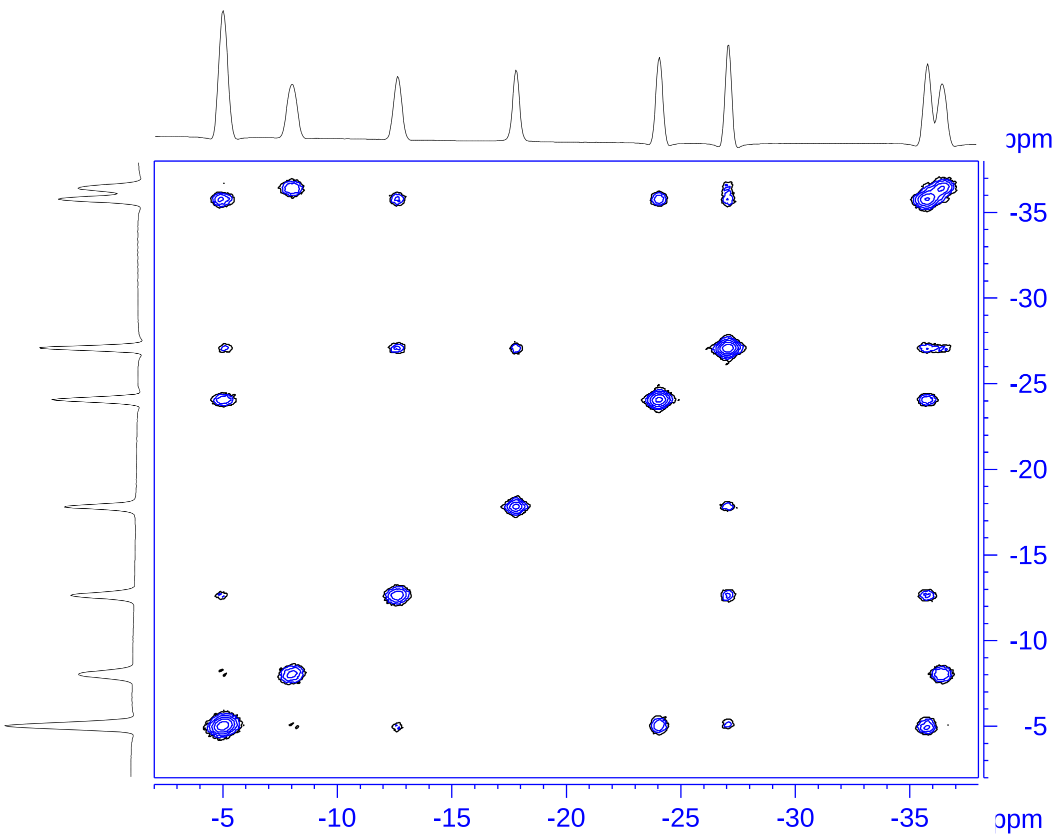

Figure S17. 2D ^{11}B-^{11}B COSY spectrum of compound **2** ($CDCl_3$, 160 MHz)

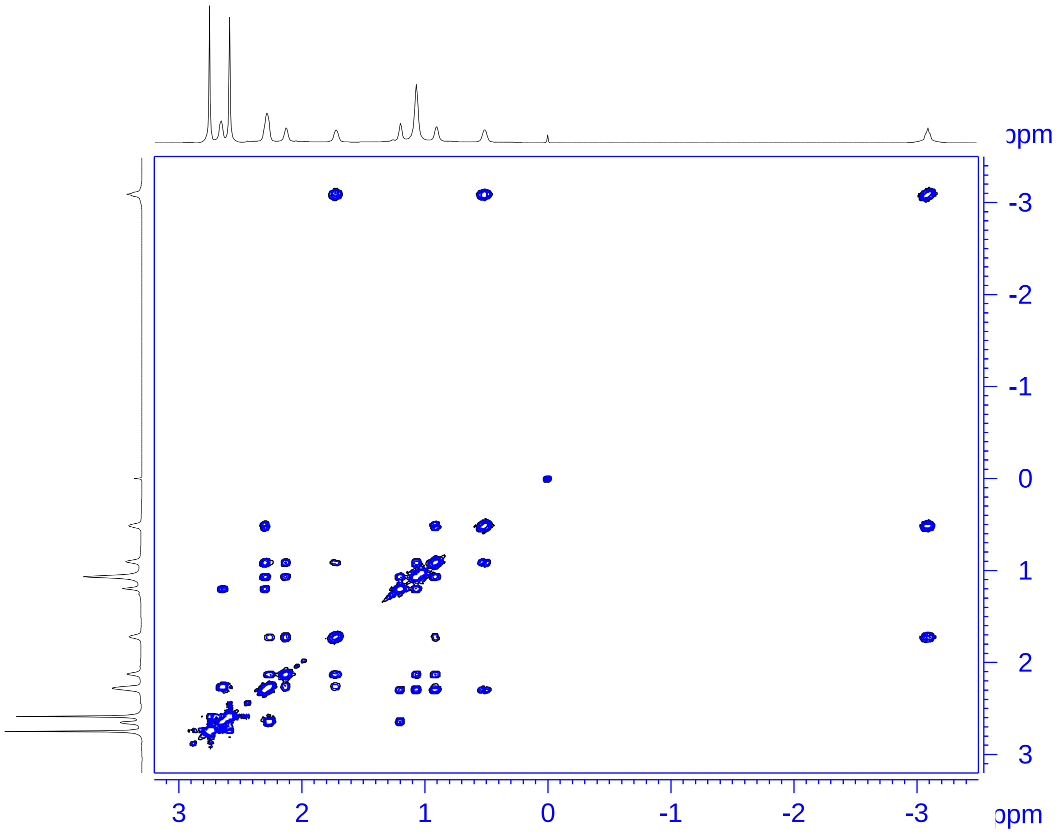

Figure S18. 2D ^{1}H-^{1}H{^{11}B} COSY spectrum of compound **2** ($CDCl_3$, 500 MHz)

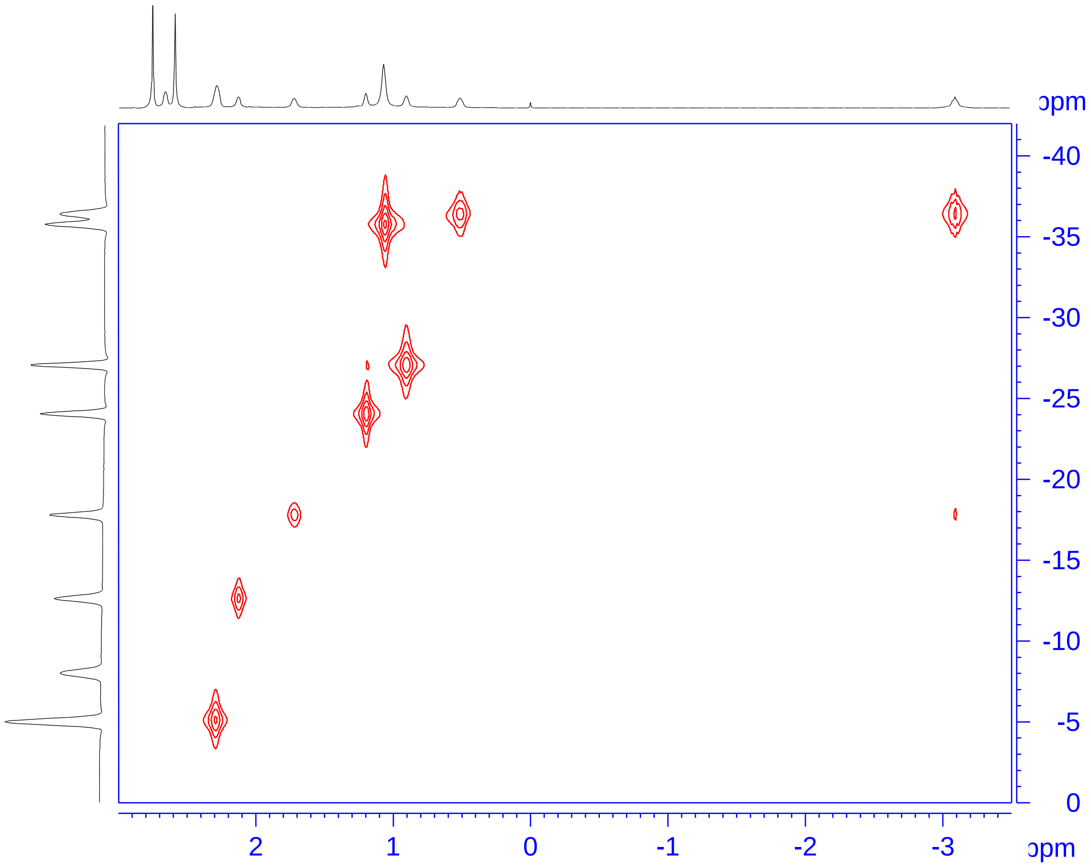

Figure S19. 2D $^{1}H\{^{11}B\}$-^{11}B HMQC spectrum of compound **2** ($CDCl_3$, 500 MHz)

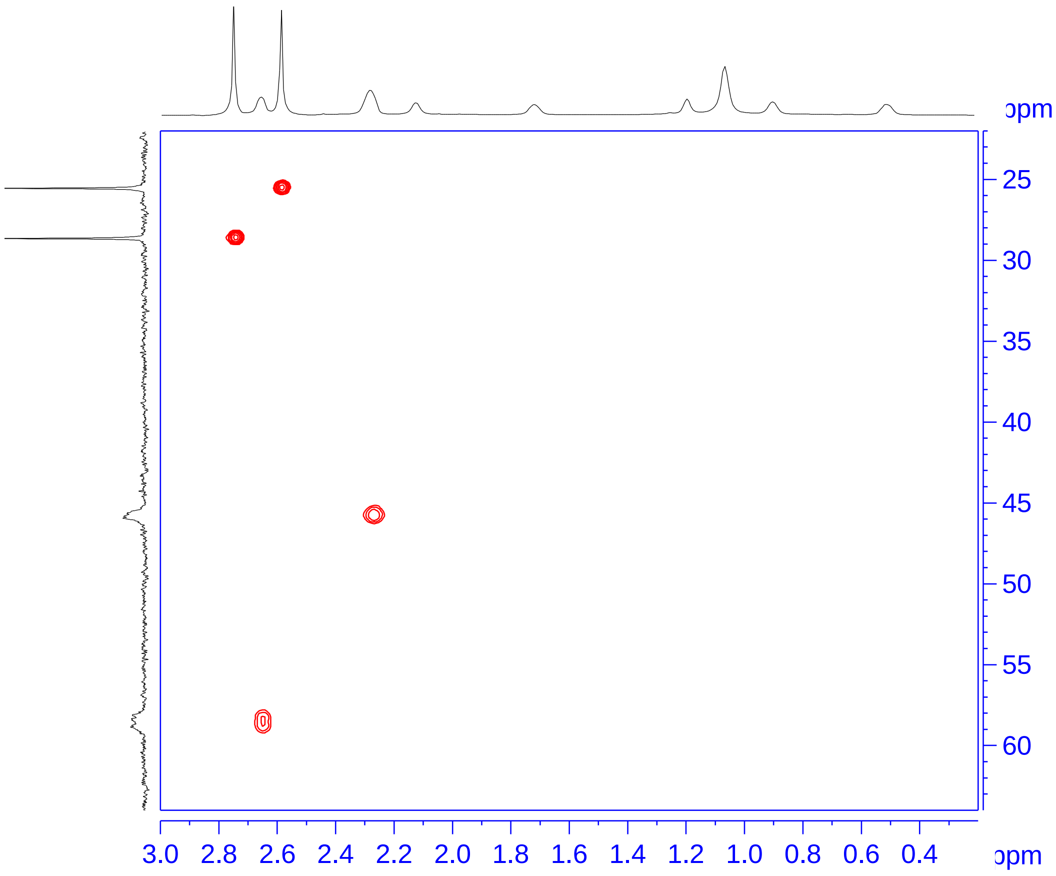

Figure S20. 2D ^{1}H-^{13}C HSQC spectrum of compound **2** ($CDCl_3$, 500 MHz)

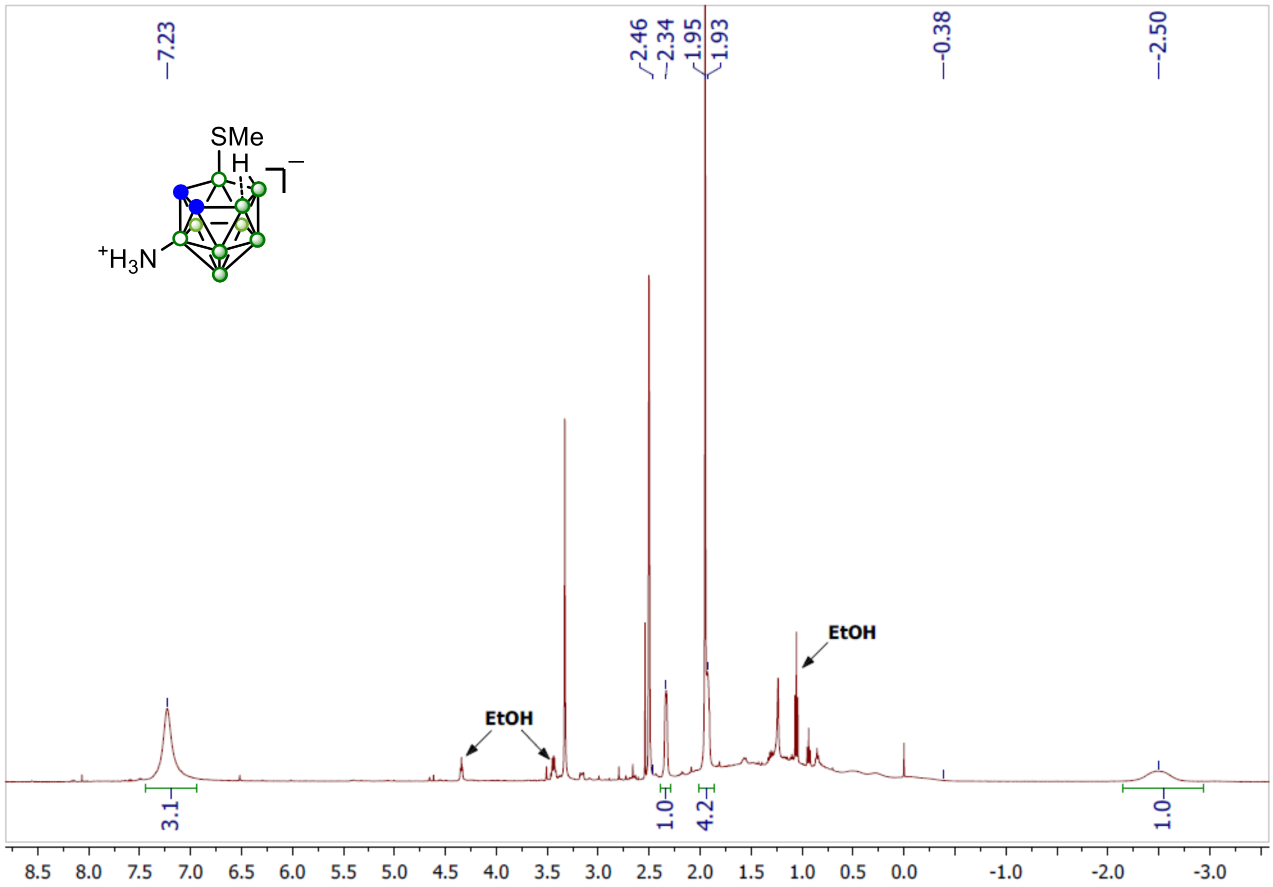

Figure S21. ^{1}H NMR spectrum of compound **6** (DMSO-d_6, 500 MHz)

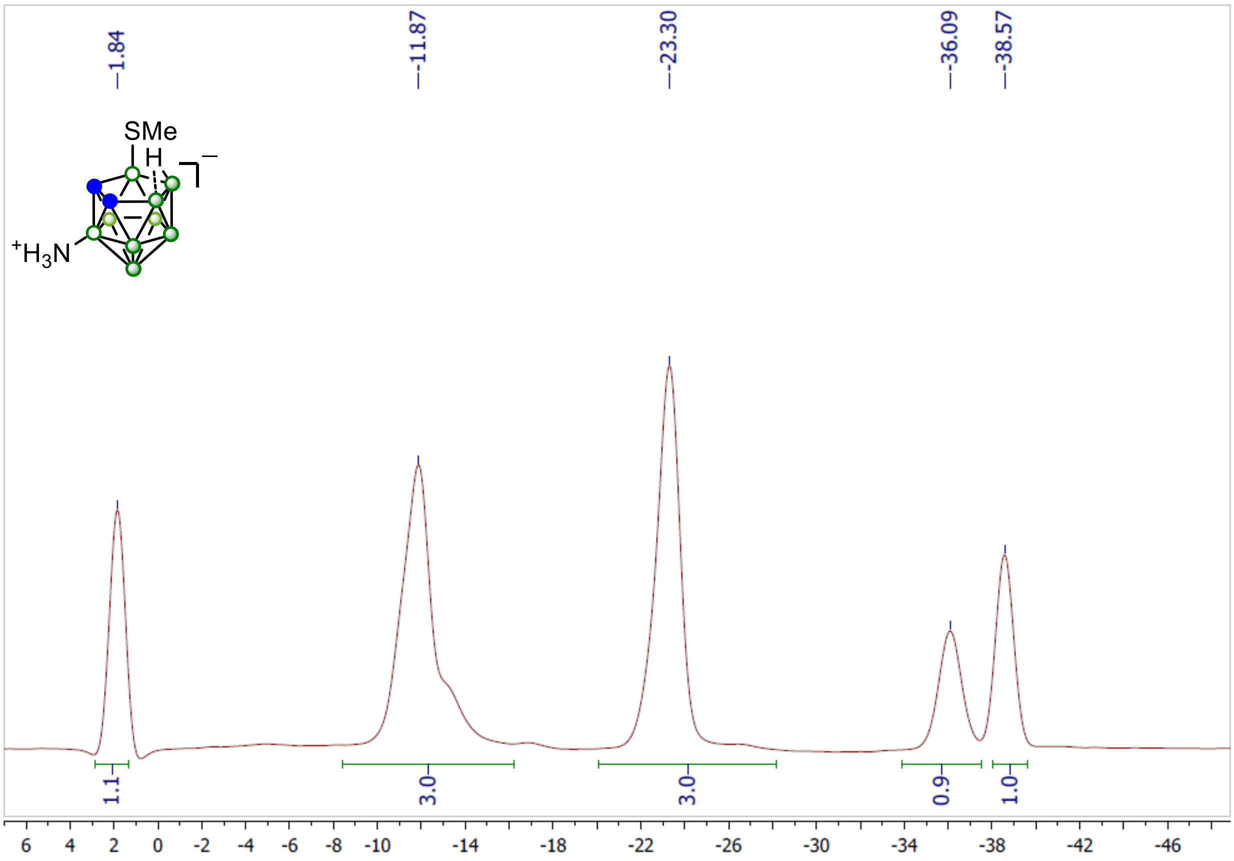

Figure S22. ^{11}B{^{1}H} NMR spectrum of compound **6** (DMSO-d_6, 128 MHz)

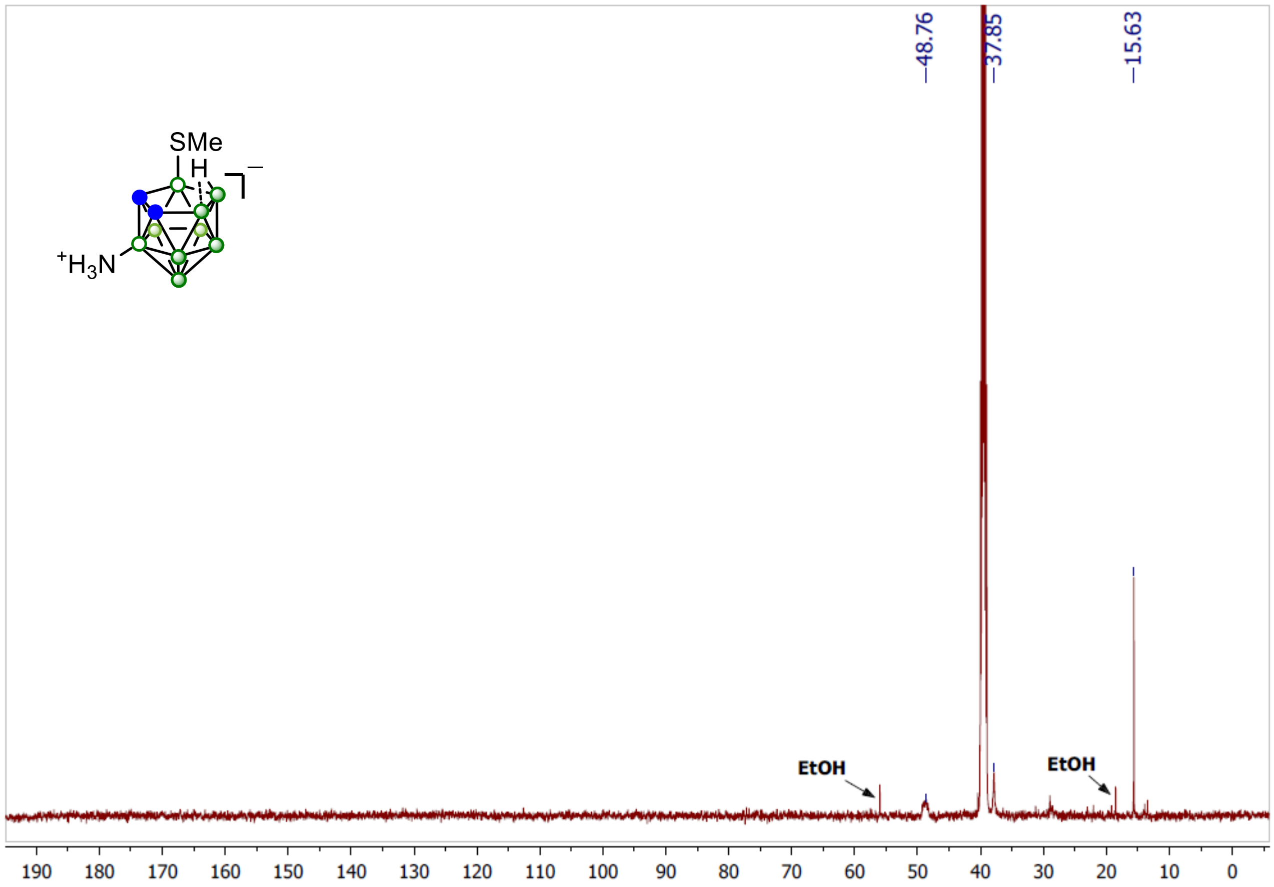

Figure S23. ^{13}C NMR spectrum of compound **6** (DMSO-d_6, 126 MHz)

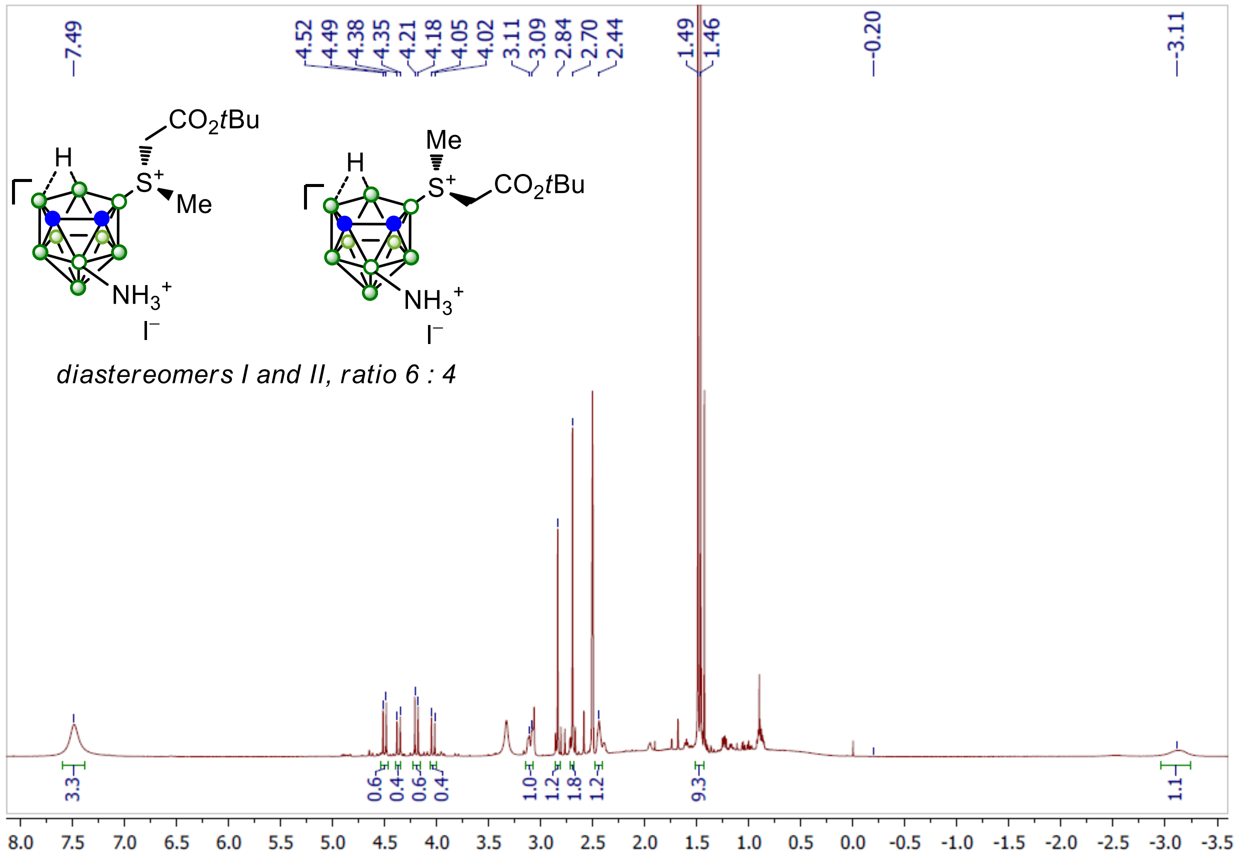

Figure S24. ^{1}H NMR spectrum of compound **7** (DMSO-d_6, 500 MHz)

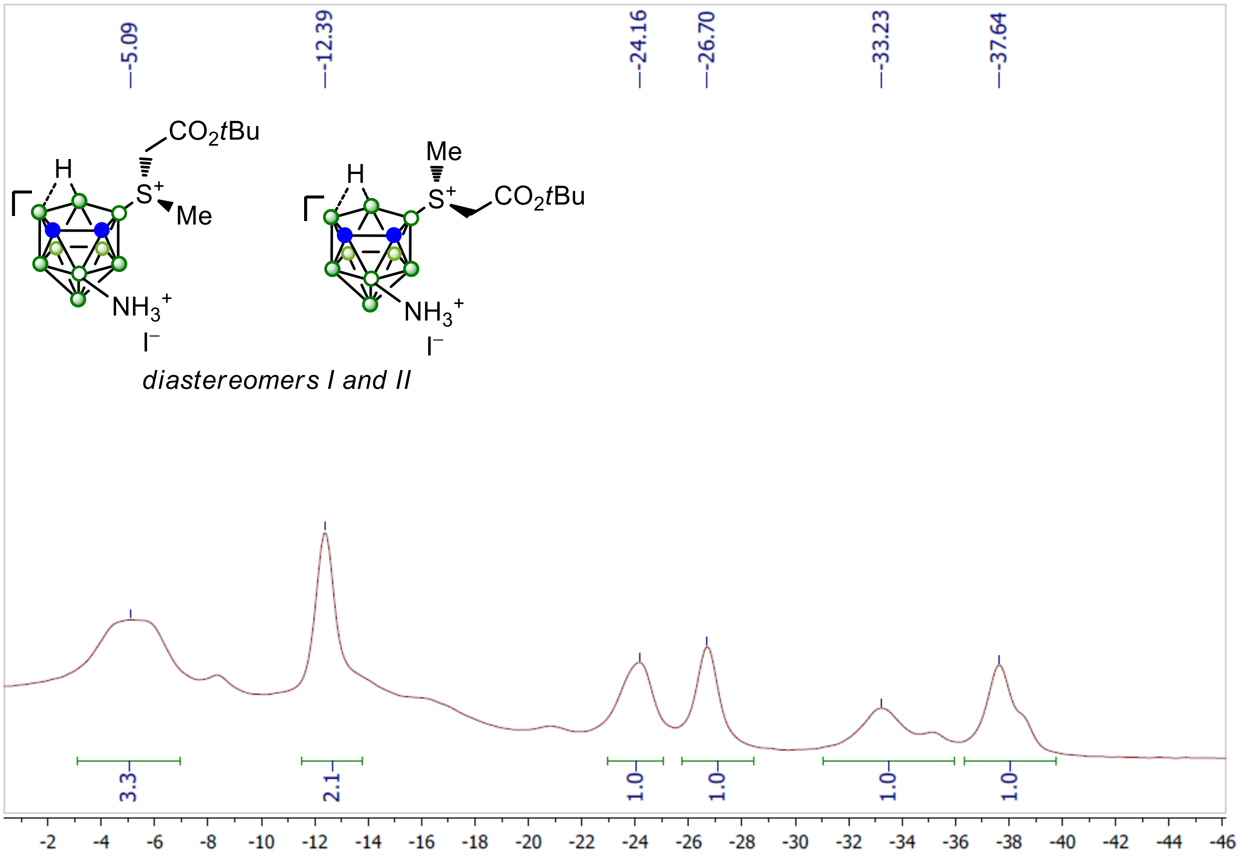

Figure S25. $^{11}B\{^1H\}$ NMR spectrum of compound **7** (DMSO-d_6, 160 MHz)

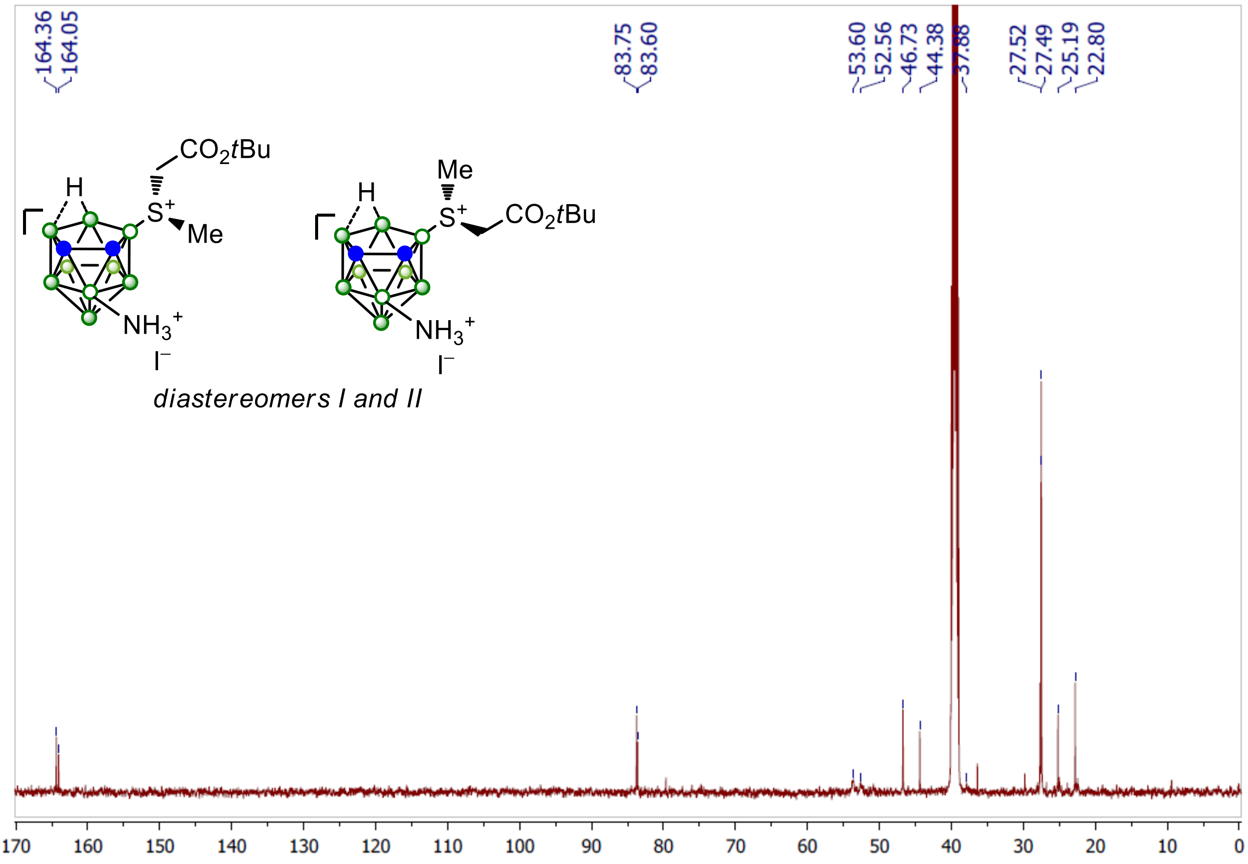

Figure S26. ^{13}C NMR spectrum of compound **7** (DMSO-d_6, 126 MHz)

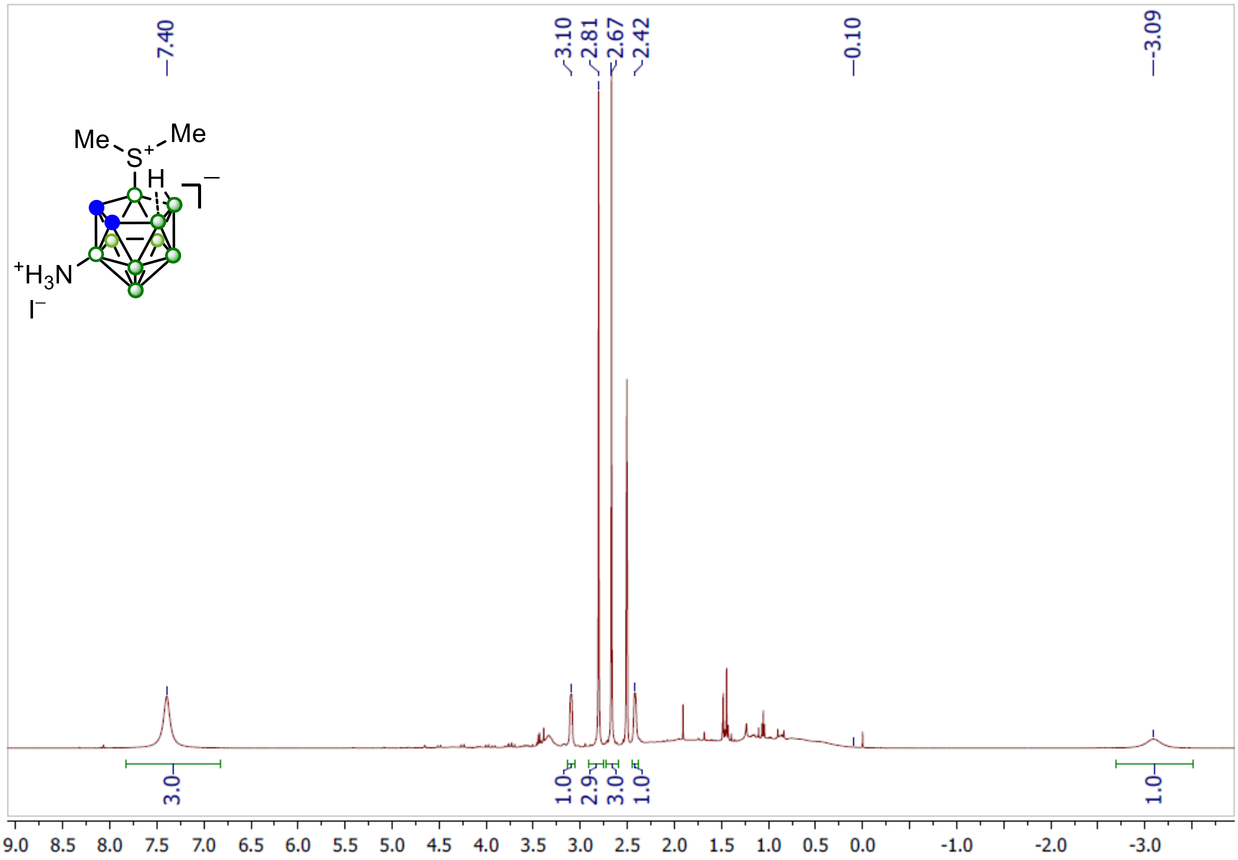

Figure S27. ^{1}H NMR spectrum of compound **2”** (DMSO-d_6, 500 MHz)

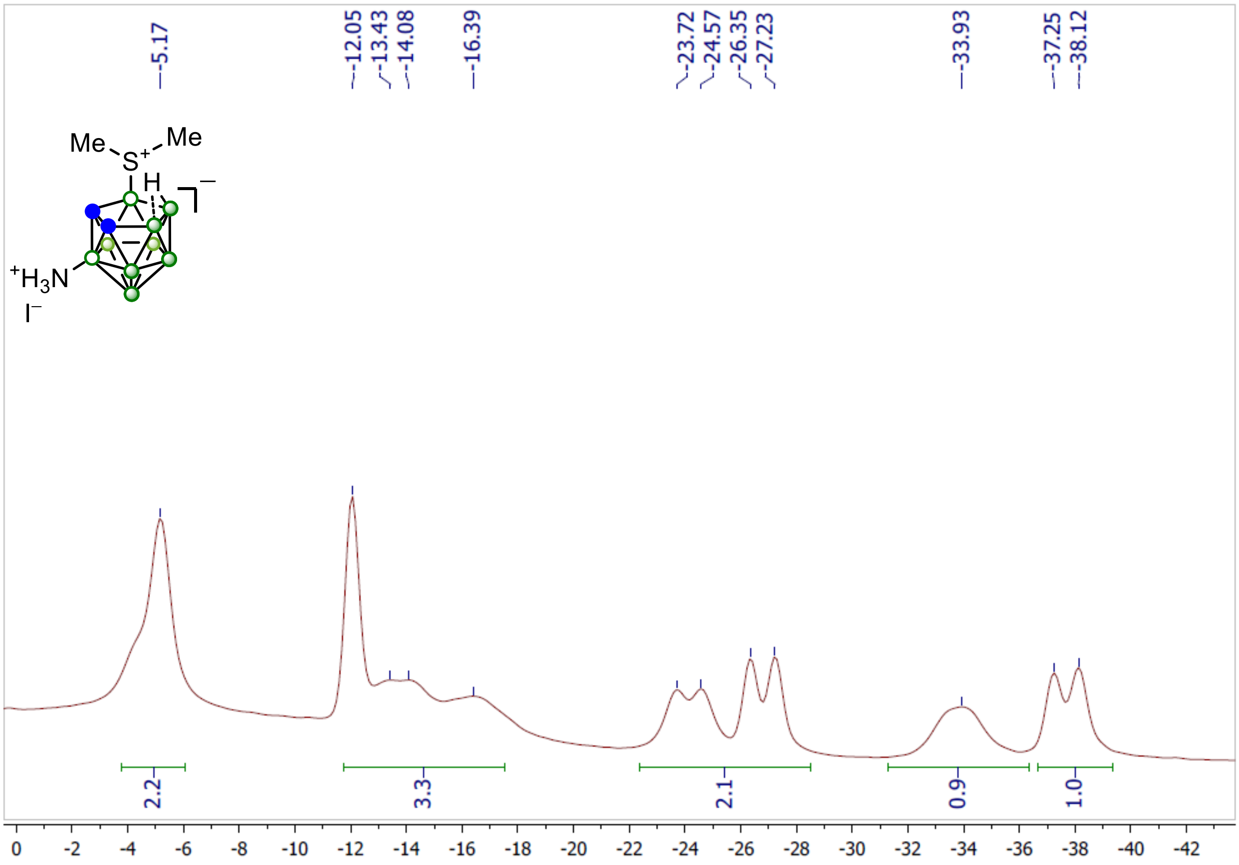

Figure S28. ^{11}B NMR spectrum of compound **2”** (DMSO-d_6, 160 MHz)

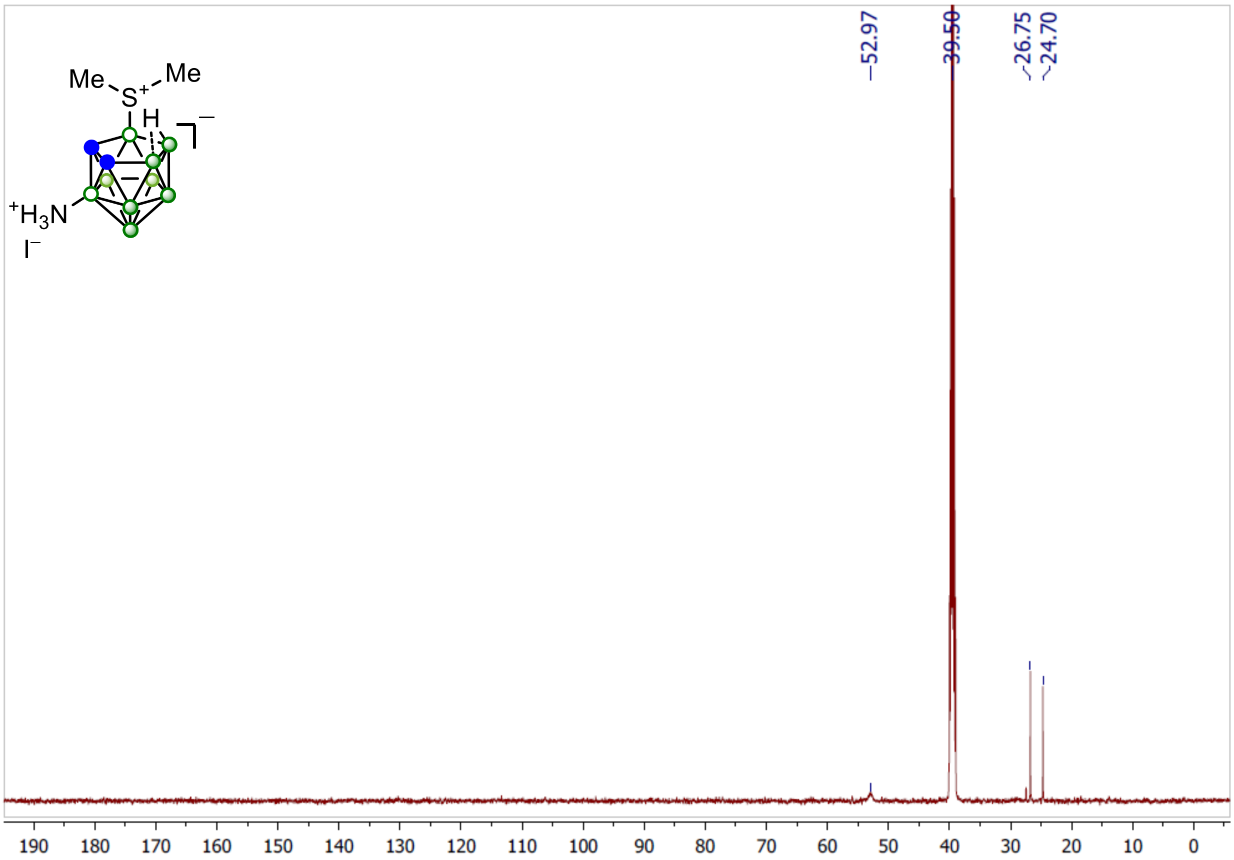

Figure S29. ^{13}C NMR spectrum of compound **2”** (DMSO-d_6, 126 MHz)

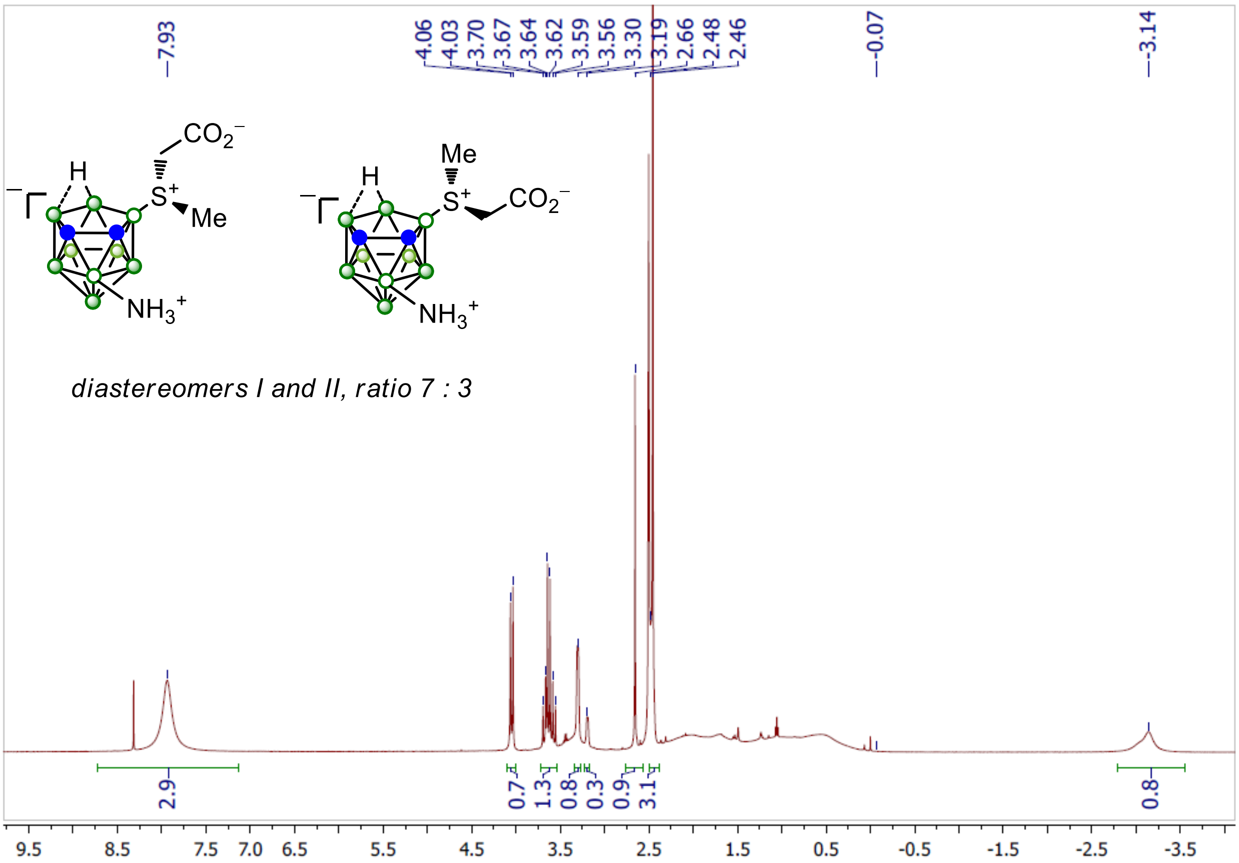

Figure S30. ^{1}H NMR spectrum of compound **1** (DMSO-d_6, 500 MHz)

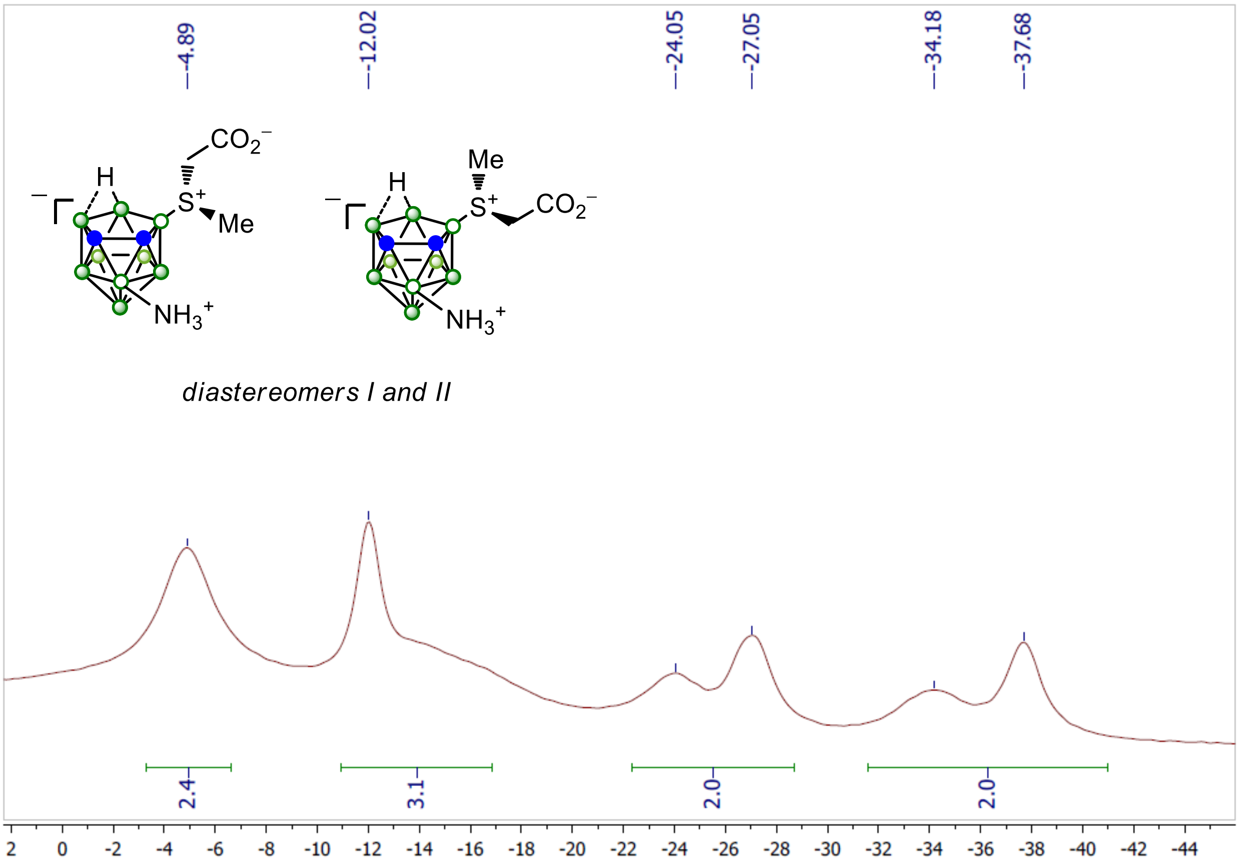

Figure S31. $^{11}B\{^{1}H\}$ NMR spectrum of compound **1** (DMSO-d_6, 160 MHz)

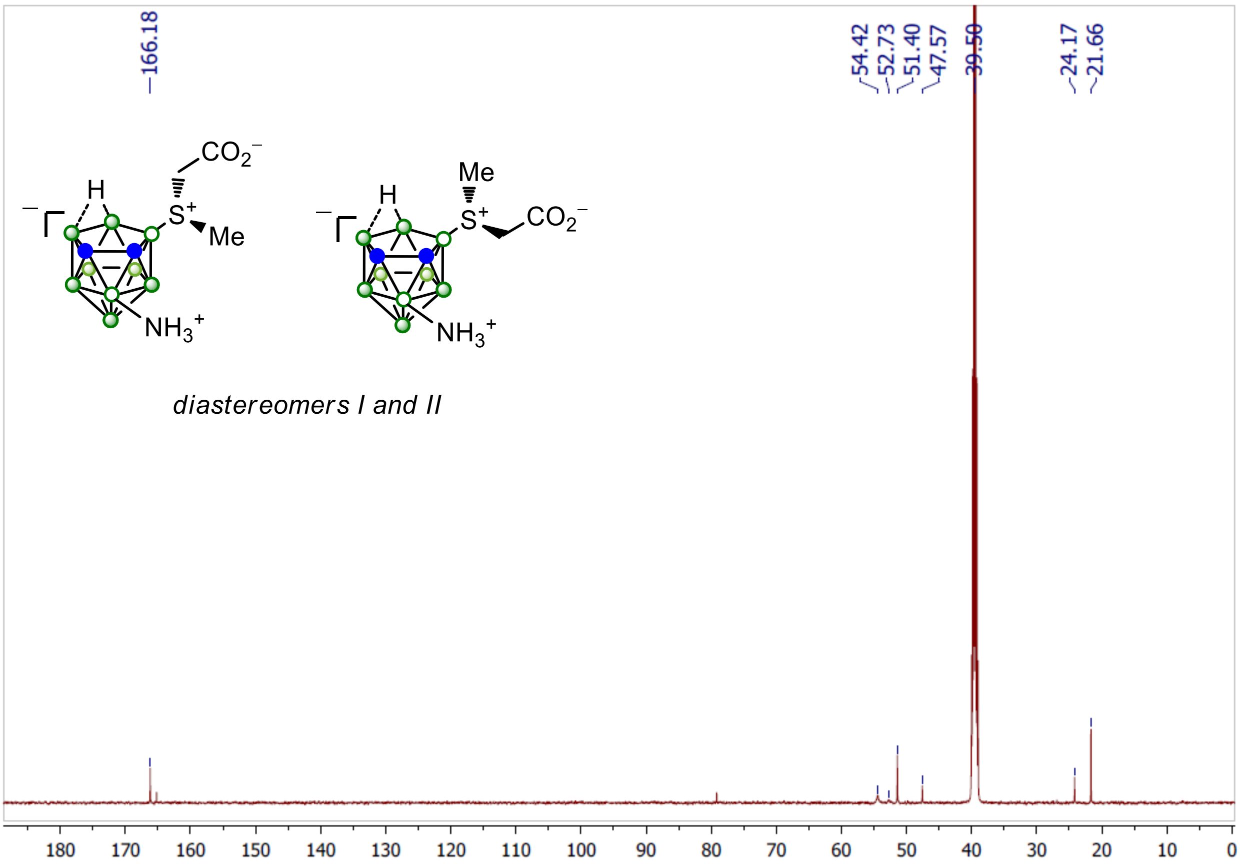

Figure S32. ^{13}C NMR spectrum of compound **1** (DMSO-d_6, 126 MHz)

HPLC Data

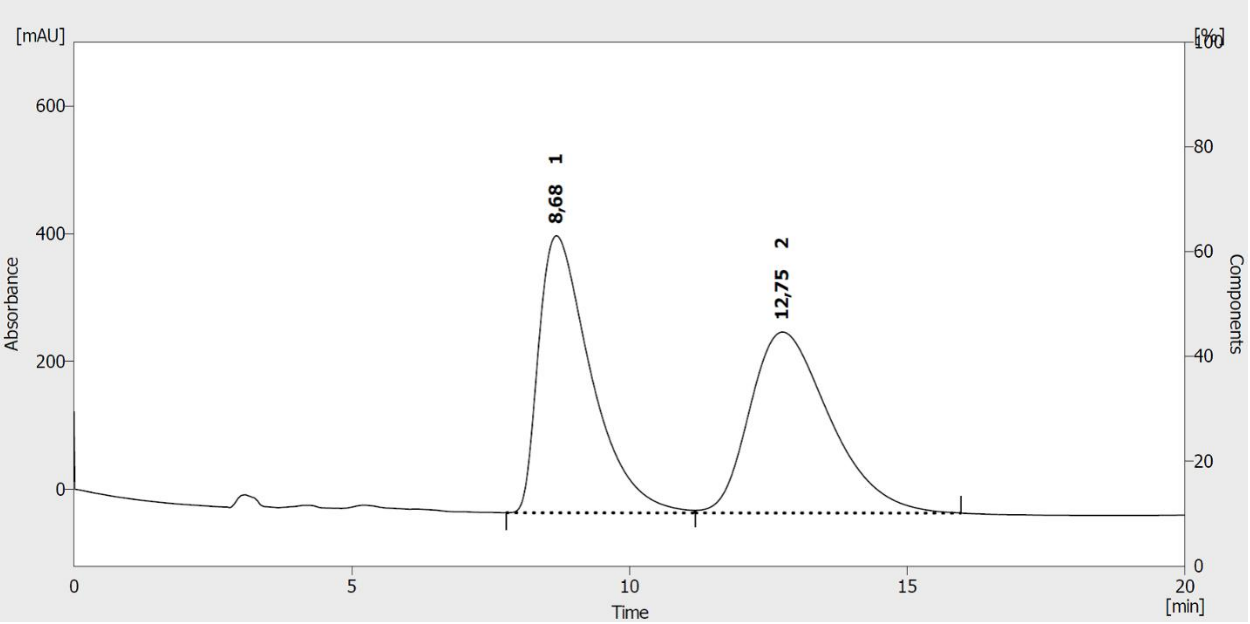

	Reten. Time [min]	Area [mAU.s]	Height [mAU]	Area [%]	Height [%]	W05 [min]
1	8,683	28440,888	433,772	50,1	60,5	1,00
2	12,750	28357,276	283,246	49,9	39,5	1,55
	Total	56798,163	717,018	100,0	100,0	

Figure S33. Chromatogram of compound **6** (Chiralpak AS-H, *n*-hexane–*i*PrOH 5:1, 1.0 mL/min, 220 nm)

HRMS Data

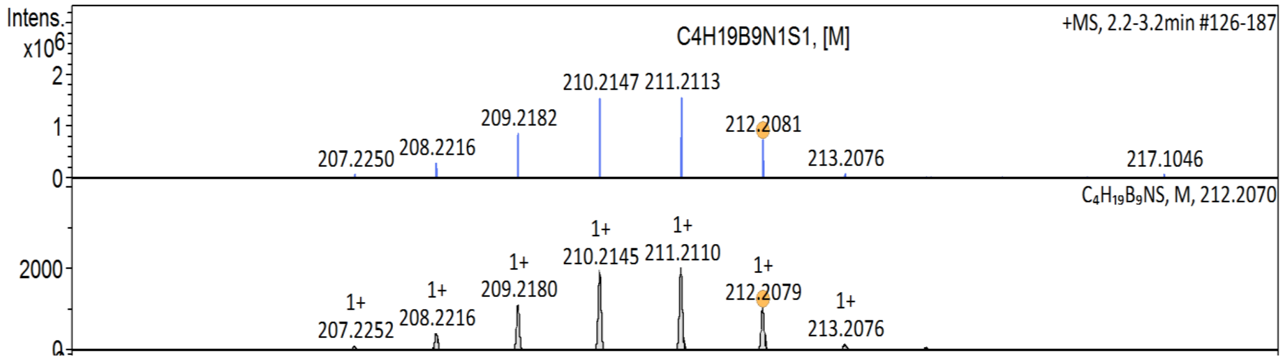

Figure S34. Experimental and simulated peak distribution in high-resolution mass spectrum of compound **2'**

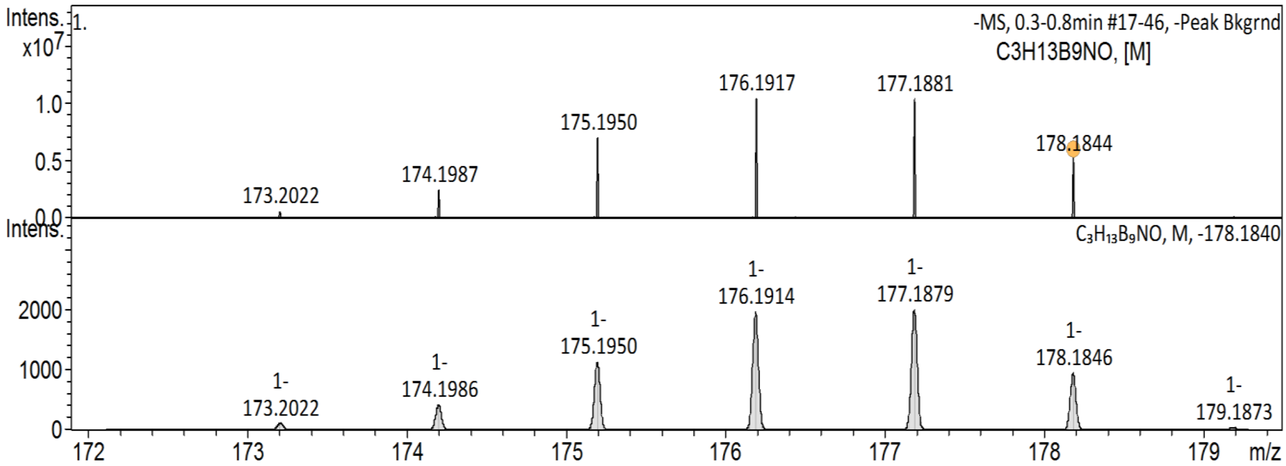

Figure S35. Experimental and simulated peak distribution in high-resolution mass spectrum of compound **5a**

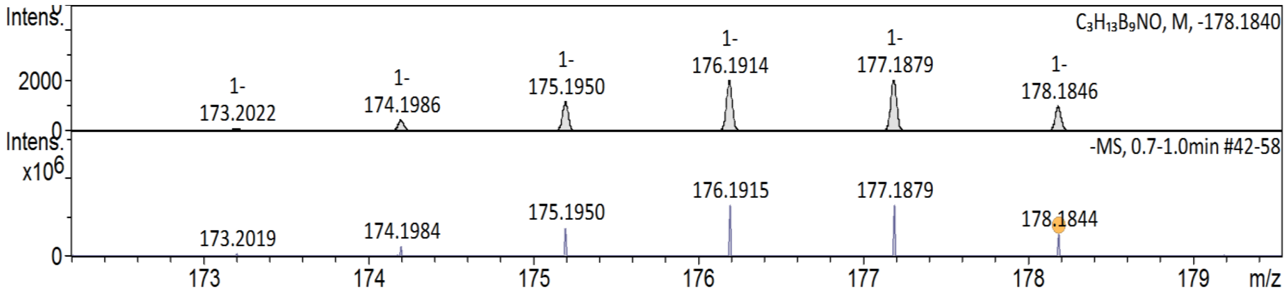

Figure S36. Experimental and simulated peak distribution in high-resolution mass spectrum of compound **5b**

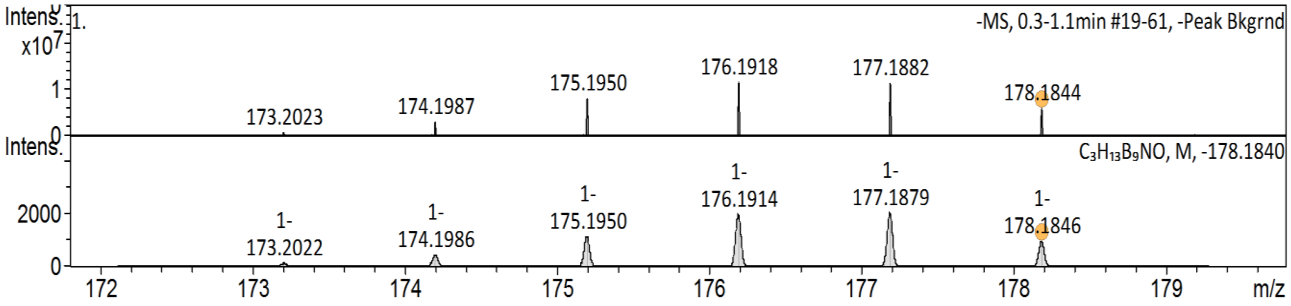

Figure S37. Experimental and simulated peak distribution in high-resolution mass spectrum of compound **5c**

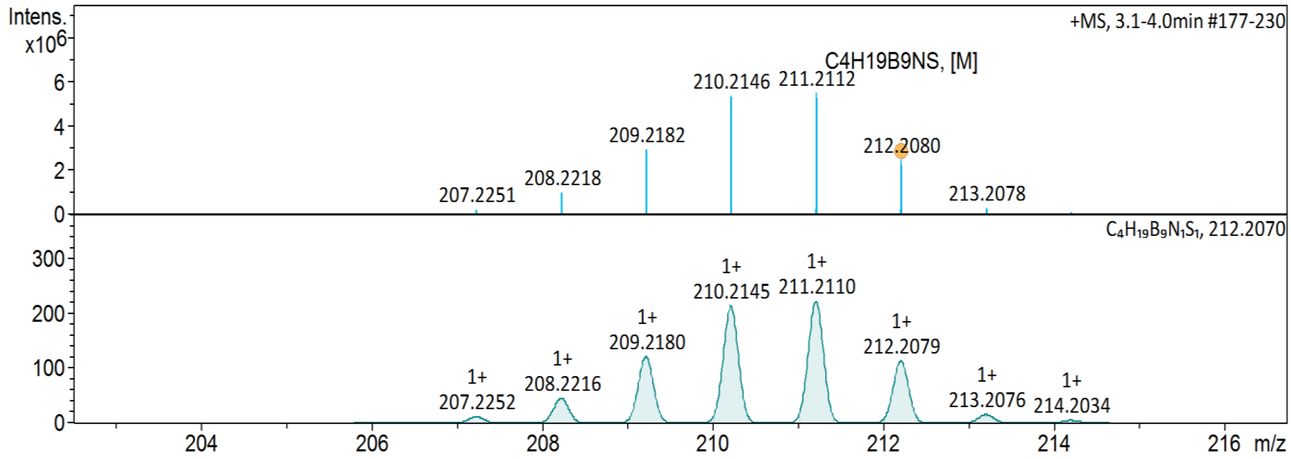

Figure S38. Experimental and simulated peak distribution in high-resolution mass spectrum of compound **2**

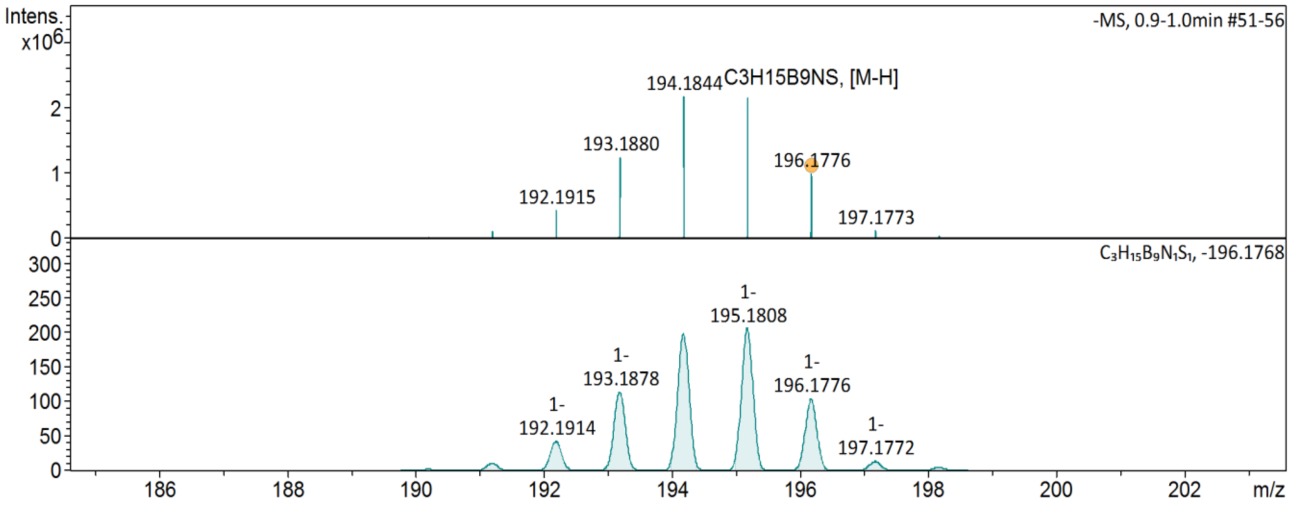

Figure S39. Experimental and simulated peak distribution in high-resolution mass spectrum of compound **6**

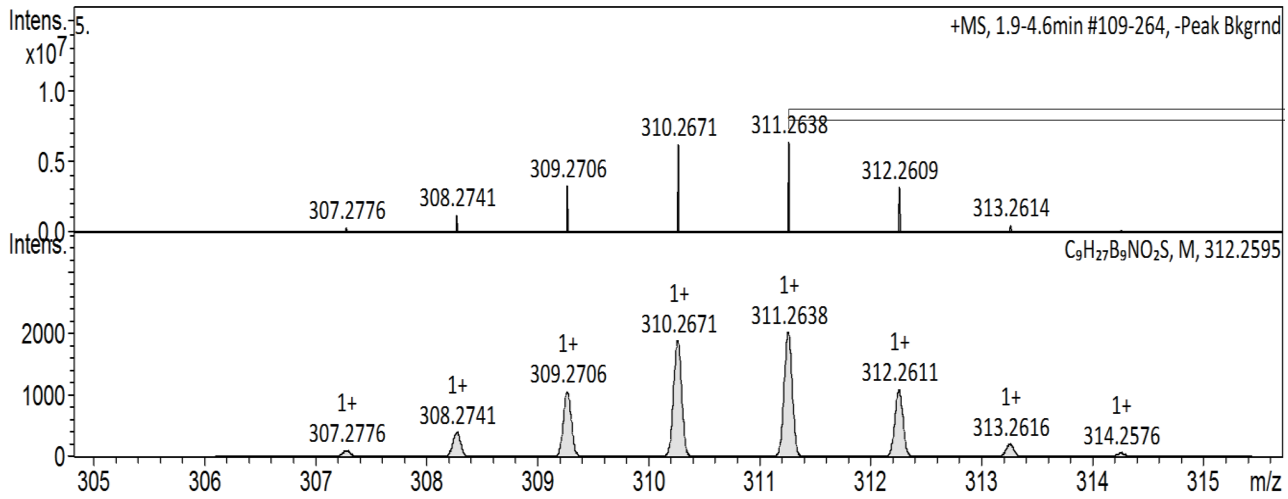

Figure S40. Experimental and simulated peak distribution in high-resolution mass spectrum of compound **7**

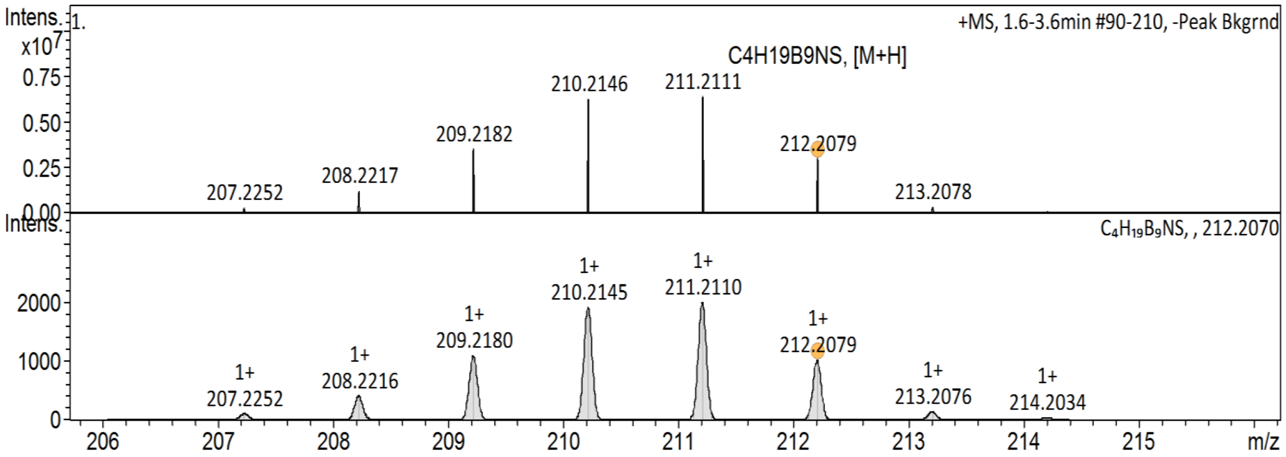

Figure S41. Experimental and simulated peak distribution in high-resolution mass spectrum of compound **2’’**

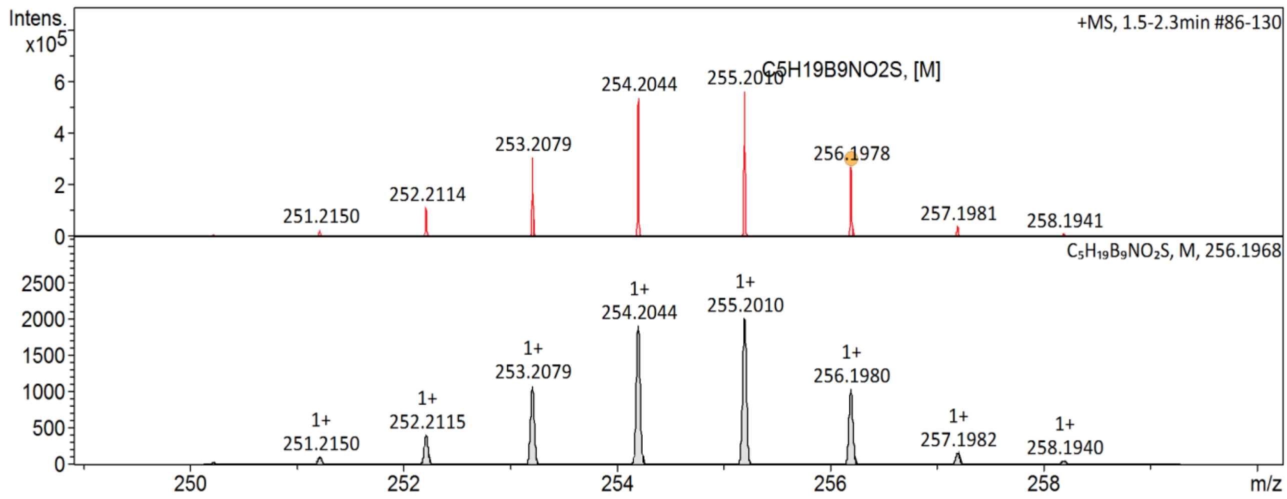

Figure S42. Experimental and simulated peak distribution in high-resolution mass spectrum of compound **1**

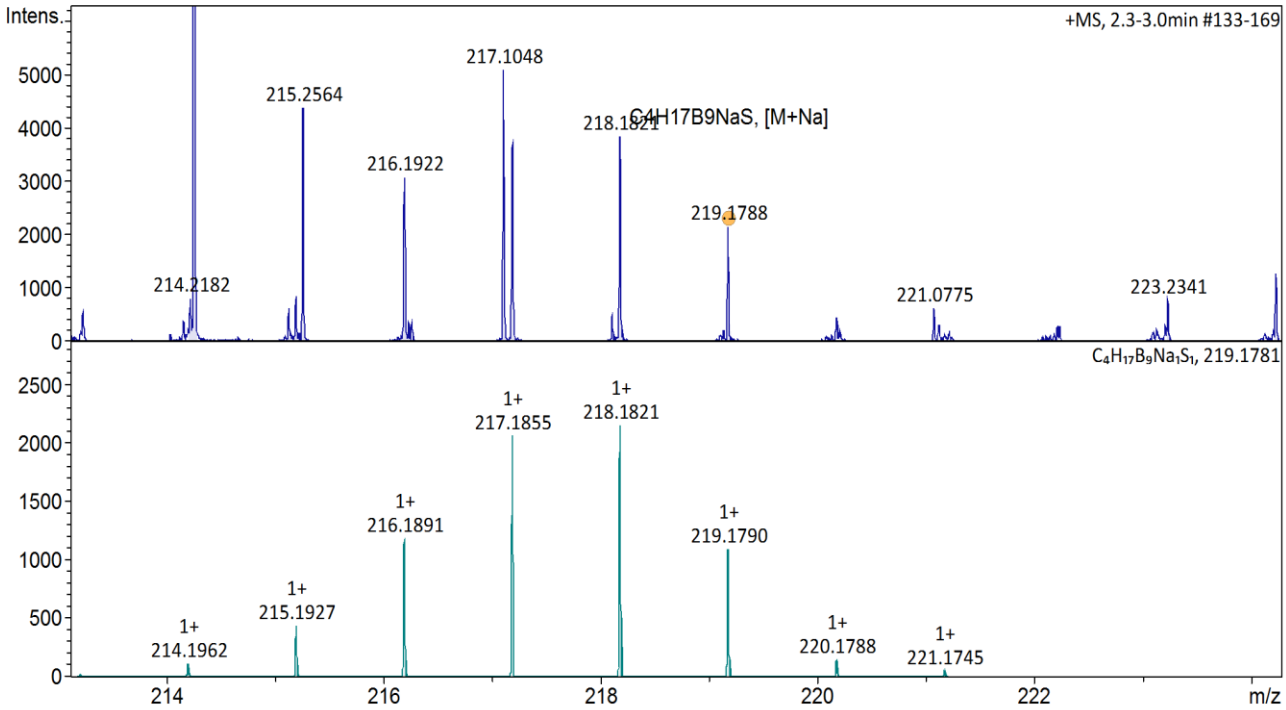

Figure S43. Experimental and simulated peak distribution in high-resolution mass spectrum of compound **9**
